# *In silico* identification and characterization of the SNPs in the human *ASTL* gene and their probable role in female infertility

**DOI:** 10.3389/fcell.2023.1151672

**Published:** 2023-06-08

**Authors:** Kapali Suri, Neha Rajput, Priya Sharma, Aishwarya D. Omble, Kiran Kulkarni, Gagandeep K. Gahlay

**Affiliations:** ^1^ Department of Molecular Biology and Biochemistry, Guru Nanak Dev University, Amritsar, Punjab, India; ^2^ Division of Biochemical Sciences, CSIR-National Chemical Laboratory, Pune, India

**Keywords:** ovastacin, ASTL, fertilization, single nucleotide polymorphism, omics, female infertility

## Abstract

Ovastacin (ASTL), a zinc metalloprotease, is released from a fertilized egg during exocytosis of cortical granules which occurs minutes after the sperm and egg fuse. ASTL cleaves ZP2, one of the four primary glycoproteins of human zona pellucida, and this cleavage prevents polyspermy, causes zona pellucida hardening, and also protects the pre-implantation embryo. Any perturbation in the activity of ASTL can thus disturb this process and may lead to infertility without changing the gross morphology of the oocyte. A small amount of ASTL is also released by unfertilized oocytes but its catalytic activity is absent as it is bound by its inhibitor, Fetuin-B (FETUB). Pre-mature release of ASTL when FETUB is absent also causes infertility. To identify and understand the structural and functional effects of deleterious SNPs of *ASTL* on its interaction with ZP2 and FETUB and hence on fertility, a total of 4,748 SNPs from the dbSNP database were evaluated using a variety of *in silico* tools. All of the 40 shortlisted nsSNPs were present in the catalytic domain of the protein. Comparison of the wild type with mutants using MutPred2 suggests an alteration in the catalytic activity/zinc binding site in many SNPs. Docking studies show the involvement of hydrophobic interactions and H bonding between ASTL and ZP2 and also between ASTL and FETUB. Four positions in ASTL involved in the hydrophobic interactions (P^105^ and D^200^ between ASTL and ZP2; D^198^ and L^278^ between ASTL and FETUB) and 5 in H bonding (E^75^ and R^159^ between ASTL and ZP2; and K^93^, R^159,^ and C^281^ between ASTL and FETUB) have SNP’s associated with them validating their importance. Interestingly, a cluster of multiple SNPs was found in the motif ^198^DRD^200^, which is also a well-conserved region among several species. Statistical Coupling Analysis (SCA) suggested that the deleterious SNPs were present in the functionally important amino acid positions of ASTL and are evolutionarily coupled. Thus, these results attempt to identify the regions in ASTL, mutations in which can affect its binding with ZP2 or FETUB and cause female infertility.

## 1 Introduction

Reproduction is an elemental biological process for the continued existence of any species. In mammals, reproduction occurs through the symphonized process of fertilization which involves the union of an oocyte and a spermatozoon to form an embryo. Mammalian oocytes are surrounded by an extracellular matrix of glycoproteins called the Zona Pellucida (ZP) that has essential roles to play before and after fertilization. These include species-specific binding to sperm, inducing acrosome reaction, preventing polyspermy, and protecting the embryo till zona hatching and implantation (Zhao and Dean, 2002; Litscher and Wassarman, 2020). Cortical reaction, a key mechanism used to prevent polyspermy, occurs minutes after the sperm and egg nuclei fuse and involves the exocytosis of cortical granules resulting in the cleavage of ZP2, one of the four ZP glycoproteins present in human oocytes. This cleavage of ZP2, which occurs at its N terminus, is brought about by a cortical granule zinc metalloprotease Ovastacin (Burkart et al., 2012). Cleaved ZP2 prevents any new sperm to bind to the fertilized egg (and thus blocks polyspermy) and also triggers zona pellucida hardening, which protects the pre-implantation embryo (Gahlay et al., 2010; Burkart et al., 2012).

**FIGURE 1:**
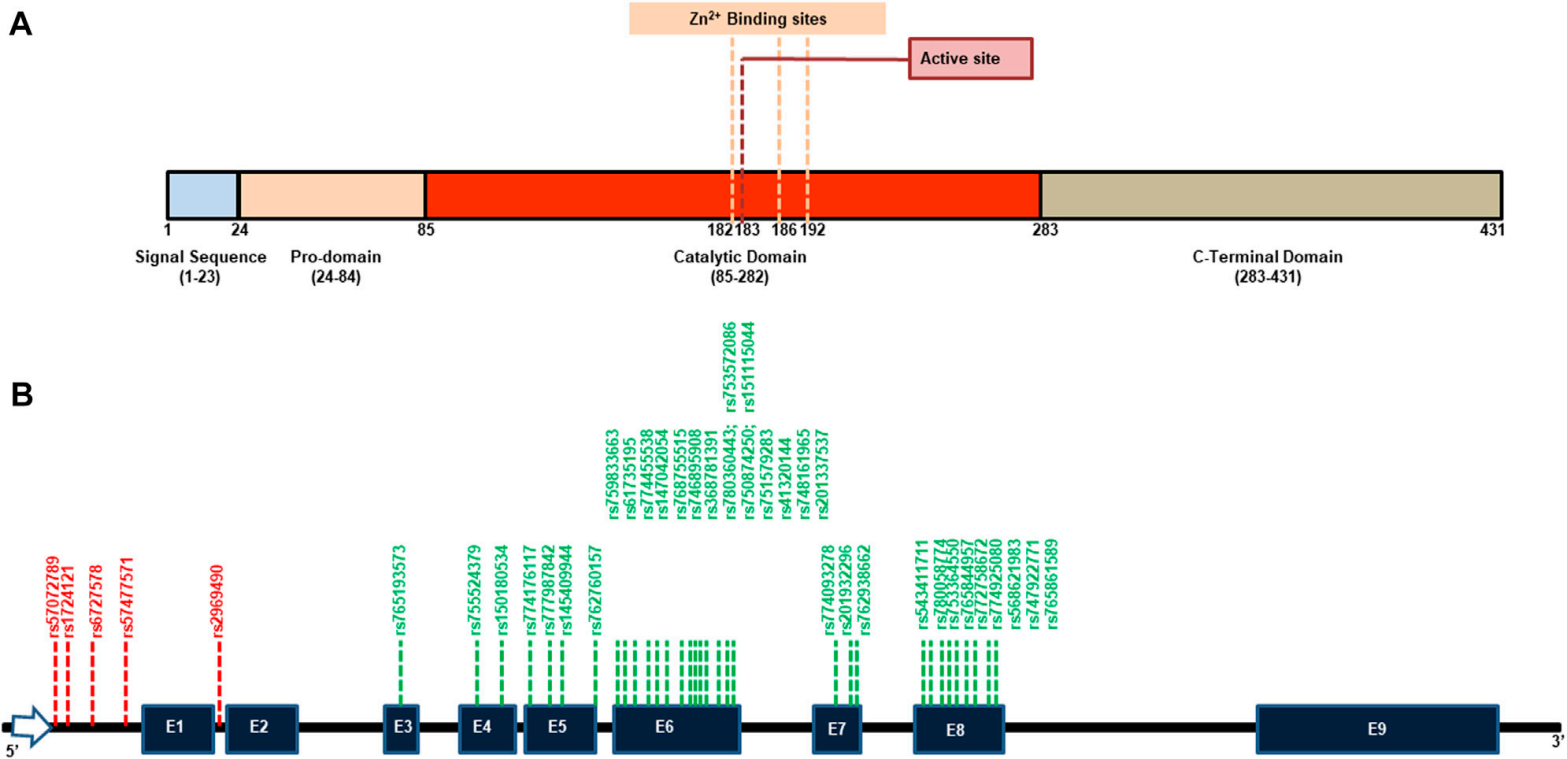
Representative domain organization and location of SNPs in human *ASTL*. **(A)** The pro-enzyme includes a secretory signal peptide (23 aa), a pro-domain (60 aa), the catalytic domain (198 aa) and a unique C-terminal domain (148 aa). The catalytic domain includes the active site Glu183 and a single zinc atom complexed to three adjacent histidine residues at position 182, 186 and 192. **(B)** A map of *ASTL* gene highlighting the positions of the various deleterious SNPs shortlisted after *in silico* analysis. Deleterious SNPs in the non-coding region are marked in red while those in the coding region are marked in green. E = Exon.

Ovastacin, a member of the astacin family of metalloproteases, is produced in oocytes and stored in cortical granules beneath the oolemma of unfertilized oocytes and requires Zn^2+^ for its activity. Human ovastacin (ASTL) exhibits the same domain organization as other astacins which includes a signal sequence, a pro-domain, a metalloprotease domain, and a C-terminal domain (Figure 1A). A localization motif present in the pro-domain (^52^DKDIPAIN^64^) targets the protein to the cortical granules via a regulated secretory pathway (Xiong et al., 2017). For the enzyme to be active, the pro-domain (24–84 aa) is cleaved creating a substrate binding cleft and making available the Zn^2+^ which otherwise was coordinated with the conserved Asp in the pro-domain. This Zn^2+^ then coordinates with the three histidine residues (in bold) adjacent to the active site Glu (^182^HELMHVLGFWH^192^) making the enzyme active (Guevara et al., 2010). The significance of ASTL in fertility is evident as *ASTL* ^−/−^ female mice exhibit a modest decrease in litter size (Burkart et al., 2012). Lack of ASTL prevents cleavage of ZP2 post fertilization which may cause polyspermy and lack of zona pellucida hardening. Polyspermic embryos do not proceed to maturation and the absence of hardened zona cannot protect the embryo, both of which may lead to decreased fertility and poor embryonic outcome. Reduced fertility was also observed when the cleavage site of ASTL on ZP2 was mutated (Gahlay et al., 2010).

Interestingly even unfertilized oocytes release small amounts of ASTL which can cause limited proteolysis of ZP2, yet definitive zona pellucida hardening is prevented because of the inhibitory effect of Fetuin-B (FETUB), a liver plasma protein present in the follicular fluid (Dietzel et al., 2013). A novel “raised-elephant-trunk” mechanism has been proposed for this inhibition (Cuppari et al., 2019). However, once cortical granule exocytosis occurs, this inhibitory action of FETUB is overcome by the release of a large amount of ASTL (Körschgen et al., 2017). Ablation of FETUB in female mice leads to infertility due to premature zona hardening of unfertilized oocytes (Dietzel et al., 2013).

Thus, changes in ASTL which can affect its binding to ZP2 or FETUB can ultimately impact female fertility. Recent research has shown that two sisters carrying a homozygous recessive truncating variant of ASTL, in which two-thirds of the catalytic protease domain and the entire C-terminal domain is removed, experienced reduced/absent fertility similar to that observed in *ASTL* ^−/−^ female mice (Maddirevula et al., 2022). The truncated protein was a result of a splice acceptor variation caused due to a c.456-1G>A transition (NM_001002036.3) in intron 5 of the ASTL gene. Multiple sperm heads and multiple pronuclei were observed in the oocytes after overnight *in vitro* fertilization and, resulted in failed IVF’s. Embryos generated via Intracytolasmic Sperm Injection (ICSI) also didn’t lead to successful pregnancy. Thus, given the potential impact of ASTL on female infertility, it becomes imperative to study changes in the *ASTL* gene which can be brought about by non-synonymous single nucleotide polymorphisms (nsSNPs) and have not yet been looked into. These nsSNPs can lead to single amino acid substitutions in a protein sequence that may affect the gene’s function and form the genetic basis of various human diseases including female infertility.

Hence, in this manuscript, SNP data for the human *ASTL* gene available from the dbSNP database was analyzed using various *in silico* tools and post-structural analysis, we have identified and predicted the most deleterious SNPs that could alter the structure, function, and stability of the protein or alter the regulation of its gene expression. These deleterious SNPs were found in the catalytic domain of the protein in functionally important amino acid positions which are evolutionarily coupled as determined by Statistical Coupling Analysis (SCA). We also predicted how these SNPs could affect the binding of ASTL with ZP2 and FETUB. This information can be useful in the diagnosis and treatment of idiopathic female infertility and in improving the outcomes of assisted reproductive technology.

## 2 Materials and methods

### 2.1 Datasets, SNP retrieval and minor allele frequency

**FIGURE 2:**
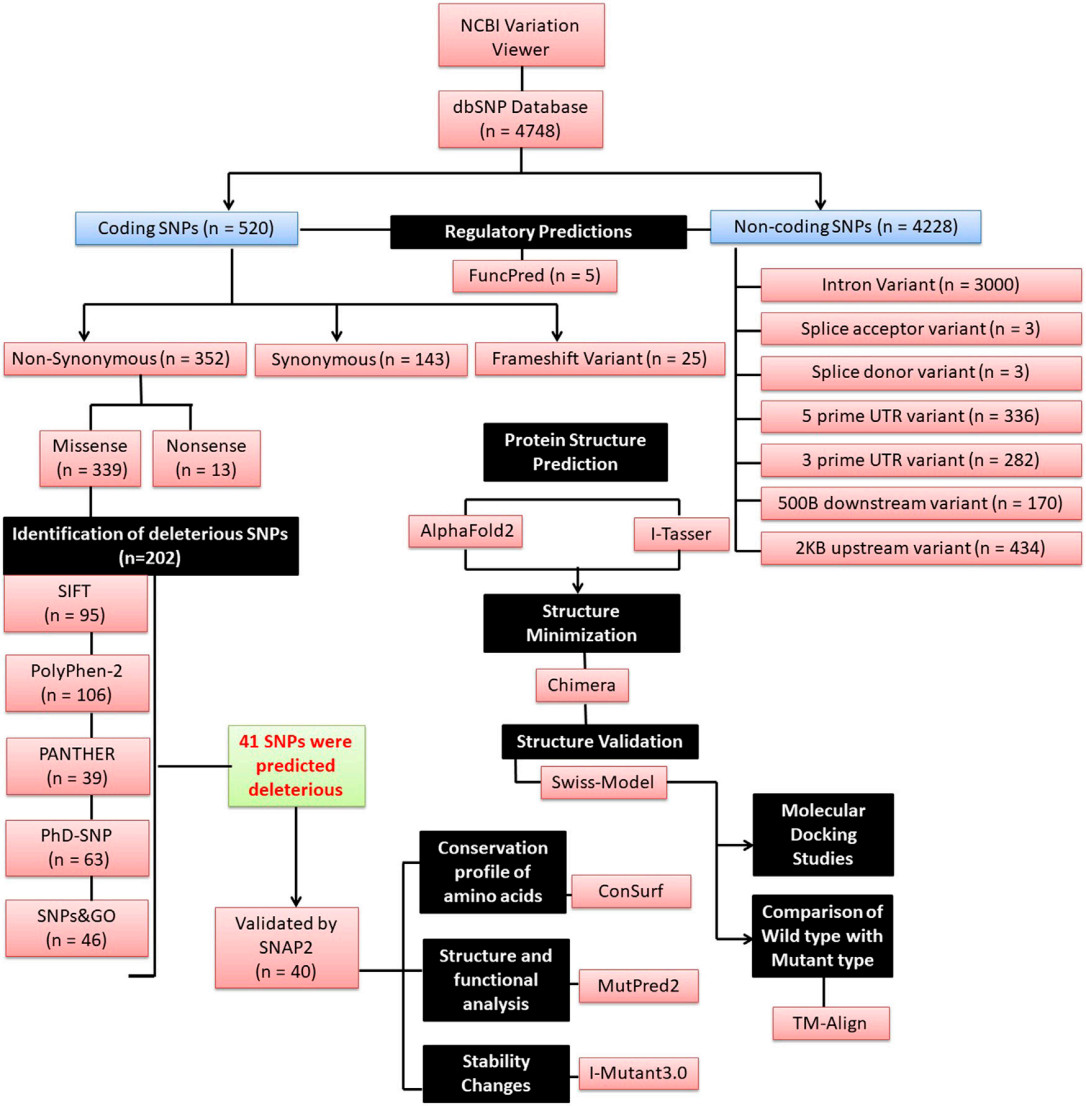
A schematic flowchart of the different *in silico* tools used (n = number of SNPs).

The information for the human Ovastacin gene (*ASTL;* NM_001002036.4) was obtained from the Variation Viewer resource at the National Center for Biological Information (NCBI) website (http://www.ncbi.nlm.nih.gov/variation/view/; Assembly: GRCh38.p14 (GCF_000001405.40); Chr 2 (NC_000002.12)). The Variation Viewer helps with browsing, finding, and viewing variations that are maintained in dbSNP, dbVar, and ClinVar in their genomic context. The SNP data for *ASTL* (rsIDs, chromosomal position, residue change) was obtained from the NCBI dbSNP database, which is a free public resource for genetic variation (collected till December 2022). SNPs validated by either 1000 Genomes Project or Exome Aggregation Consortium (ExAC) or both were collected and further divided into two categories: coding and non-coding. Furthermore, the coding SNPs were classified as non-synonymous, synonymous, and frameshift variations whereas non-coding SNPs were classified as intron variant, splice acceptor variant, splice donor variant, 5’ UTR variant, 3’ UTR variant, 500B downstream variant, and 2 KB upstream variant. The SNPs that were verified by 1000 Genomes had frequencies of 0.005, 0.005–0.01, 0.01–0.05, and ≥0.05. Those from ExAC consisted of allele counts with “Singleton” and “2–10 alleles” and with <0.001, 0.001–0.01, 0.01–0.1, and ≥0.1 frequencies. As indicated in Figure 2, the non-synonymous SNPs were further categorized as missense and non-sense variations. Only missense SNPs were subsequently submitted to a range of *in silico* methods. Various tools were employed further to comprehend SNPs that could possibly affect the structure, function, and stability of the protein.

Global or population-specific minor allele frequency (MAF) for the shortlisted missense SNPs were obtained from the NCBI dbSNP database (using either the ExAC or ALFA project in case the MAF values in ExAC were unavailable). The MAF values of the shortlisted rsIDs for the Indian population were obtained from the Indigenomes database (https://clingen.igib.res.in/indigen/) which covers genomic data from over 1,000 whole genome sequences sequenced from across India.

### 2.2 Identification of disease-linked or deleterious nsSNPs

Firstly, five different bioinformatics tools were used to sieve out the detrimental nsSNPs. These include SIFT (Sorting Intolerant From Tolerant; https://sift.bii.a-star.edu.sg) (Sim et al., 2012); PolyPhen-2 (Polymorphism phenotyping v2; https://genetics.bwh.harvard.edu/pph2/) (Adzhubei et al., 2013), PANTHER (Protein Analysis Through Evolutionary Relationships; http://pantherdb.org/) (Mi et al., 2013), PhD-SNP (Predictor of human Deleterious Single Nucleotide Polymorphisms; http://snps.biofold.org/phd-snp/phd-snp) (Capriotti and Fariselli, 2017) and SNPs&GO (https://snps.biofold.org/snps-and-go/) (Capriotti et al., 2013). Later the SNPs that had been predicted to be potentially deleterious by at least 4 tools out of the 5 (SIFT, PolyPhen-2, PANTHER, PhD-SNP, and SNPs&GO)) were selected for further validation with SNAP2 (Screening for Non-Acceptable Polymorphism; https://rostlab.org/services/snap/) (Hecht et al., 2015). All of these tools use different principles such as alignment score (SIFT), naïve Bayes classifier (PolyPhen-2), hidden Markov model (PANTHER) neural network (SNAP2), or support vector machine (PhD-SNP and SNPs&GO) to determine if a non-synonymous single nucleotide change in the coding region was deleterious or benign.

### 2.3 Structural and functional changes in ASTL caused by deleterious nsSNPs

MutPred2 (http://mutpred.mutdb.org), a widely used machine learning approach, was utilized to find pathogenic variants as well as the structural and functional changes caused by detrimental nsSNPs (Pejaver et al., 2020). It forecasts their impact on more than 50 different protein properties, allowing it to classify them as pathogenic or benign. Protein sequence in FASTA format along with a list of amino acid substitutions in the corresponding FASTA headers was used as input. A *p*-value threshold of 0.05 and a prediction score ranging between 0 and 1 was used with a higher score reflecting a higher probability of pathogenicity. The possible alterations in properties were represented as gain/loss of protein structure and/or function.

### 2.4 Predicting the effect of nsSNPs on the stability of ASTL

I-Mutant3.0 (http://gpcr2.biocomp.unibo.it/cgi/predictors/I-Mutant3.0/I-Mutant3.0.cgi/) was used to predict a change in the stability of ASTL upon a single nucleotide change (Capriotti et al., 2005). The related shift in free energy (Kcal/mol; expressed as DDG) and stability (Reliability Index; RI; spanning from 0 to 10) were calculated for the native ASTL as well as the various SNPs shortlisted above. A RI value of 10 represents the highest reliability.

### 2.5 Evolutionary conservation profile of ASTL

The ability to identify important mutations requires knowledge of the evolution conservation score. Evolutionarily conserved regions are of structural and/or functional importance. ConSurf (https://consurf.tau.ac.il) tool was employed to determine the conservation score for each amino acid which is a relative measure of evolutionary conservation at each position (Ashkenazy et al., 2016). The location in a protein (DNA/RNA) that is most conserved has the lowest score. The continuous conservation scores were partitioned into a discrete scale of nine bins, such that bin 9 contains the most conserved positions and bin 1 contains the most variable positions, and then color grades were assigned to facilitate graphic visualization. For ASTL, the layers used for assigning grades were: from −1.552 to −1.207 (grade 9), from −1.207 to −0.862 (grade 8), from −0.862 to −0.517 (grade 7), from −0.517 to −0.172 (grade 6), from −0.172 to 0.292 (grade 5), from 0.292 to 0.875 (grade 4), from 0.875 to 1.458 (grade 3), from 1.458 to 2.041 (grade 2) and from 2.041 to 2.624 (grade 1).

### 2.6 Structure prediction, minimization, and quality assessment of protein structure

I-TASSER (https://seq2fun.dcmb.med.umich.edu//I-TASSER/) (Zhou et al., 2022) and AlphaFold2 (https://colab.research.google.com/github/sokrypton/ColabFold/blob/main/AlphaFold2.ipynb#scrollTo=kOblAo-xetgx) (Jumper et al., 2021) was used to create a 3D model for ASTL. Model ranked as 1 in AlphaFold2, based on the highest per-residue confidence score (pLDDT score; values above 90 imply strong confidence, whereas those below 50 denote poor confidence), was selected. For I-TASSER, the model which had the highest C-score was selected. Both the models were optimized and minimized using a protein preparation wizard of Chimera 1.16 (Goddard et al., 2005). Thereafter, using the Swiss-model structure assessment tool (https://swissmodel.expasy.org/assess) (Kiefer et al., 2009), the models were compared based on several parameters such as QMEAN for Quality Estimation (in an ideal case the value must be as low as possible), MolProbity for structure-validation (value as low as possible) and Ramachandran plot to visualize energetically favored regions for backbone dihedral angles of protein structure (in an ideal case, amino acids under the favored region of Ramachandran plot should be >98%, outliers <0.2%, bad bonds = 0, clash score = 0, bad angle = 0) and sequence similarity. The best model was then chosen for further analysis. Similarly, the AlphaFold model for human ZP2 (Uniprot ID: Q05996) and human FETUB (Uniprot ID: Q9UGM5) was also downloaded from UniProt and prepared using Chimera 1.16.

### 2.7 Comparison of the structures of wild type and mutant ASTL through 3D protein modeling

To observe the deleterious effect of nsSNPs, all the substitutions were manually replaced using the rotamer function input and any new network of contacts or clashes produced were visualized in Chimera 1.16. TM-align (https://zhanglab.ccmb.med.umich.edu/TM-align/) tool was used in sequence-independent protein structure comparison. It is used for calculating TM-score and Root mean square deviation (RMSD) values. The TM-score, which ranges from 0 to 1, reveals the topological similarity between wild type and mutant models (Zhang and Skolnick, 2005). A score of one indicates a perfect match between two structures. On the other hand, the RMSD measures the average distance between the alpha-carbon backbones of wild type and mutant models and illustrates how different the mutant structure is from the wild type. Greater variance is indicated by a higher RMSD value.

### 2.8 Molecular docking studies to identify the interactions between ASTL and ZP2 as well as ASTL and FETUB

Protein-protein docking studies were performed for ASTL and ZP2 as well as ASTL and FETUB using HADDOCK (High Ambiguity Driven protein-protein DOCKing). HADDOCK sets itself apart from *ab initio* docking techniques by using ambiguous interaction restraints (AIRs) to carry out the docking process using data from known or expected protein interfaces (Dominguez et al., 2003). It is used for a wide class of protein-protein, protein-nucleic acids, and protein-ligand complexes. The human ASTL catalytic domain (85–282 aa) and the human ZP2 region from 40–370 aa corresponding to the N-terminal domain were docked. For ASTL-FETUB docking, the catalytic domain of ASTL was docked with full-length FETUB.

### 2.9 Prediction of SNPs affecting gene regulation

Out of a total of 4,748 SNPs reported in the dbSNP database, only 774 SNPs (coding as well as non-coding) were submitted to FuncPred (SNP Function Prediction) (https://snpinfo.niehs.nih.gov/snpinfo/snpfunc.html) to detect their role in the regulation of gene expression like splicing sites, Transcription factor binding sites (TFBS), microRNA binding sites, etc. (Xu and Taylor, 2009).

### 2.10 Statistical coupling analysis of ASTL proteins

Curated ASTL proteins were downloaded from the pBLAST search and aligned using CLUSTALW. SCA was performed using the python scripts provided on https://reynoldsk.github.io/pySCA/ (Rivoire et al., 2016).

## 3 Results

### 3.1 Retrieval of SNP dataset from dbSNP database

A total of 4,748 SNPs for the *ASTL* gene were found in the NCBI dbSNP database. 520 SNPs were detected in the coding region, of which 352 were non-synonymous variations, 143 were synonymous and 25 were frameshift. Variations in the non-coding region included intron variant (n = 3,000), splice acceptor variant (n = 3), splice donor variant (n = 3), 5’ UTR variant (n = 336), 3’ UTR variant (n = 282), 500B downstream variant (n = 170), and 2 KB upstream variant (n = 434).

### 3.2 nsSNPs were predicted to be deleterious in *ASTL*

Regulatory region SNPs and nsSNPs are expected to have a huge impact on the protein’s phenotype and function. Amongst the nsSNPs, only missense SNPs (n = 339) which had been validated by either 1000 Genomes or ExAC or by both (n = 202) were evaluated by 5 different algorithms to predict the harmful effect of a single amino acid change. SIFT (SIFT score of ≤0.05) predicted 95 nsSNPs to be harmful of which 50 had the most damaging score (Score = 0; Supplementary Table S1) while PolyPhen-2 predicted 106 nsSNPs as probably/possibly damaging. Using position-specific evolutionary preservation (PSEP) score, PANTHER predicted a total of 39 nsSNPs to be probably damaging (Supplementary Table S1). PhD-SNP prediction identified 63 nsSNPs as disease-associated (Supplementary Table S1) while SNPs&GO classified 46 nsSNPs to be deleterious. 41 out of 202 nsSNPs were shortlisted based on the criteria that they were categorized as deleterious in at least four or more of the aforementioned five SNP prediction tools by manual concordance and thus likely to affect the function of the protein (Table 1; Supplementary Table S1). A different classifier named SNAP2 was used to cross-validate if these were harmful nsSNPs or just neutral variants. Only one variant rs749378316 was predicted to have neutral effect and was not further evaluated. Thus, 40 out of 41 nsSNPs were shortlisted for further analysis. All these SNPs were found to be present in the catalytic domain of the protein (Figure 1).

**TABLE 1:** List of the shortlisted 41 deleterious nsSNPs found in the *ASTL* gene which were further evaluated by SNAP2.

S. No.	Variant ID	Substitution	SNAP2	S. No.	Variant ID	Substitution	SNAP2
1	rs765193573	E75K	Effect	21	rs151115044	R199W	Effect
2	rs755524379	K93N	Effect	22	rs751579283	D200N	Effect
3	rs150180534	P105L	Effect	23	rs751579283	D200Y	Effect
4	rs150180534	P105R	Effect	24	rs143172084	R204C	Effect
5	rs774176117	R117H	Effect	25	rs748161965	E209K	Effect
6	rs777987842	T131M	Effect	26	rs201337537	I210F	Effect
7	rs145409944	F135S	Effect	27	rs774093278	Y230C	Effect
8	rs762760157	G152R	Effect	28	rs201932296	H237Y	Effect
9	rs759833663	S155L	Effect	29	rs762938662	Y238C	Effect
10	rs61735195	R159H	Effect	30	rs762938662	Y238S	Effect
11	rs774455538	G162E	Effect	31	rs543411711	R246C	Effect
12	rs147042054	H182L	Effect	32	rs749378316	R246H	Neutral
13	rs147042054	H182R	Effect	33	rs780058774	P249L	Effect
14	rs768755515	L188P	Effect	34	rs780058774	P249R	Effect
15	rs746895908	E193K	Effect	35	rs753364550	G262S	Effect
16	rs368781391	R196W	Effect	36	rs765844957	Q263R	Effect
17	rs780360443	D198E	Effect	37	rs772758672	R264Q	Effect
18	rs753572086	D198N	Effect	38	rs774925080	L267P	Effect
19	rs753572086	D198Y	Effect	39	rs568621983	R274W	Effect
20	rs750874250	R199Q	Effect	40	rs747922771	L278P	Effect
				41	rs765861589	C281W	Effect

**FIGURE 3:**
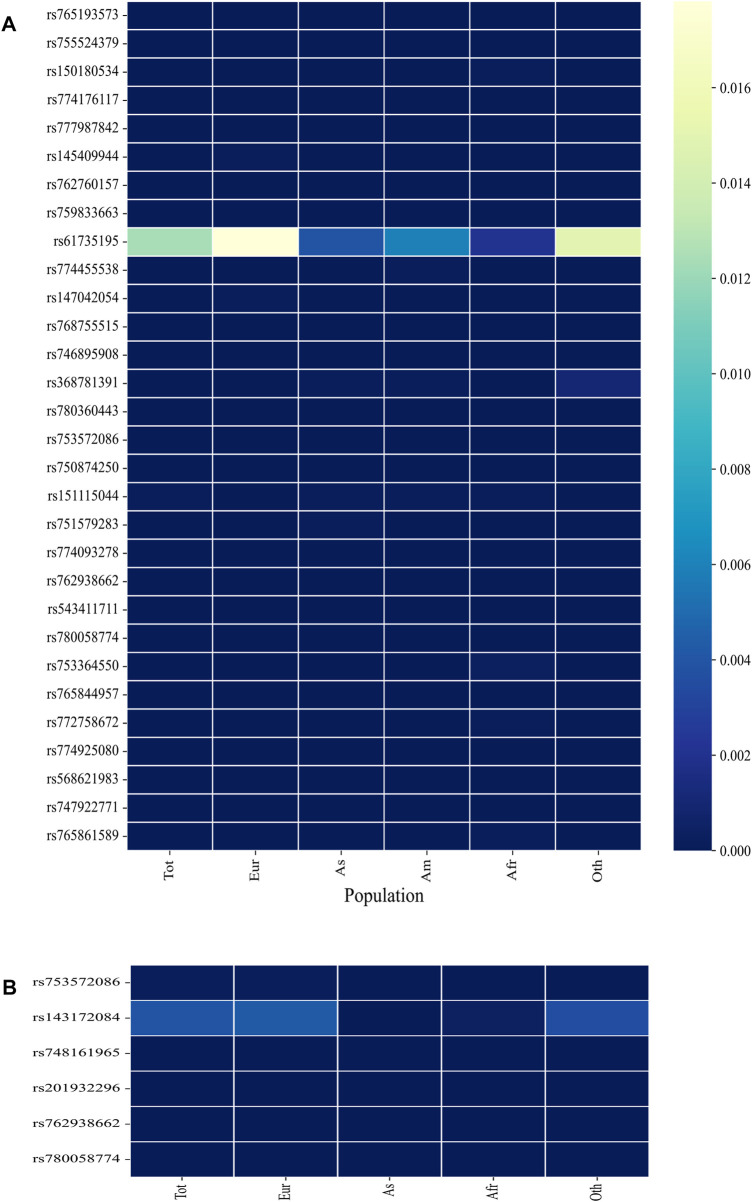
Heatmaps depicting the Minor allele frequency (MAF) of the shortlisted SNPs in the **(A)** ExAC and **(B)** ALFA database. **(B)** includes only those rsIDs whose MAF data was unavailable in ExAC. Tot = Total; Eur = European; As = Asian; Am = American; Afr = African; Oth = Other.

The minor allele frequency (MAF) of a SNP is a measure of its frequency in a population. SNPs with MAF <0.01 are considered rare variants, and if a SNP is deleterious, it is expected to have a low MAF value. A low global MAF value of <0.01 was observed for all SNPs except rs61735195 (global MAF = 0.012433; Figure 3; Supplementary Table S2). In the Indian population, only rs61735195 (MAF = 0.0063) has been described in the Indigenomes database, whereas the other shortlisted SNPs have not yet been deposited or annotated.

### 3.3 Prediction of the functional and structural effects of deleterious nsSNPs on ASTL protein and its stability

The MutPred 2 webserver predicted 36 out of the shortlisted 40 deleterious nsSNPs to cause structural and functional changes as they scored higher than 0.50 (Supplementary Table S3). These changes affected properties like altered transmembrane protein, protein stability, metal binding sites, loss of catalytic and allosteric sites, increased or decreased relative solvent accessibility, loss of disulfide linkages, altered disordered interfaces and signal peptides etc. Furthermore, I-Mutant 3.0 predicted the effect of these mutations on the stability of ASTL. A decrease in stability was observed for 34 out of the 40 nsSNPs. The resultant free energy change (kcal/mol) and the reliability index for each of the substitutions are shown in Supplementary Table S4.

### 3.4 ConSurf score prediction for predicted 41 nsSNPs

**FIGURE 4:**
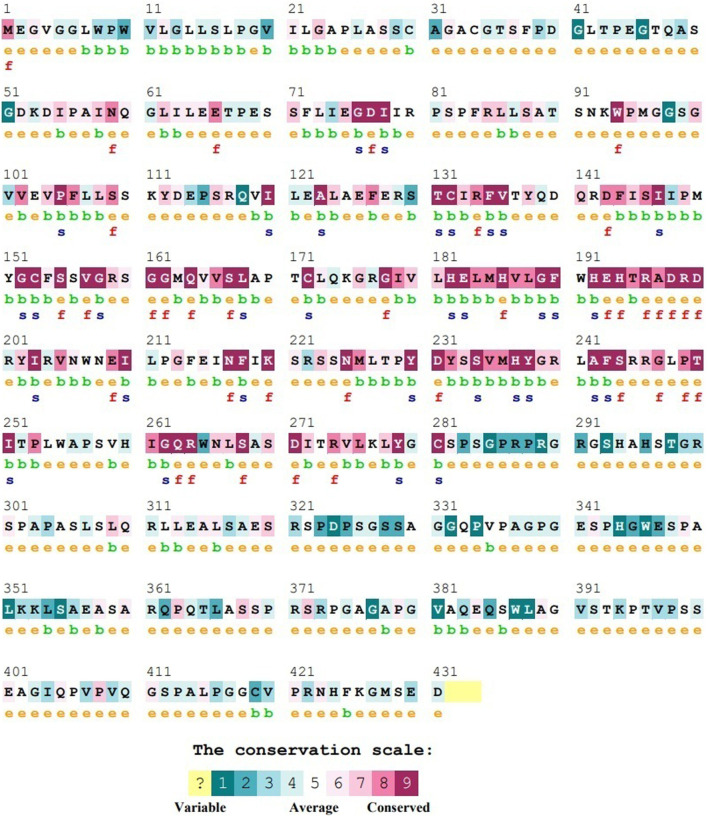
Representative figure of ConSurf output for ASTL wherein the conservation profile of the various amino acids is represented as a color bar. e: an exposed residue; b: a buried residue; f: a predicted functional residue (highly conserved and exposed); s: a predicted structural residue (highly conserved and buried).

Consurf predicted 32 out of the 41 nsSNPs to be highly conserved (Figure 4; Supplementary Table S5). All of these SNPs were present in the catalytic domain of the protein and included residues which were predicted to have either functional or structural role.

### 3.5 Homology modeling and structure validation

**FIGURE 5:**
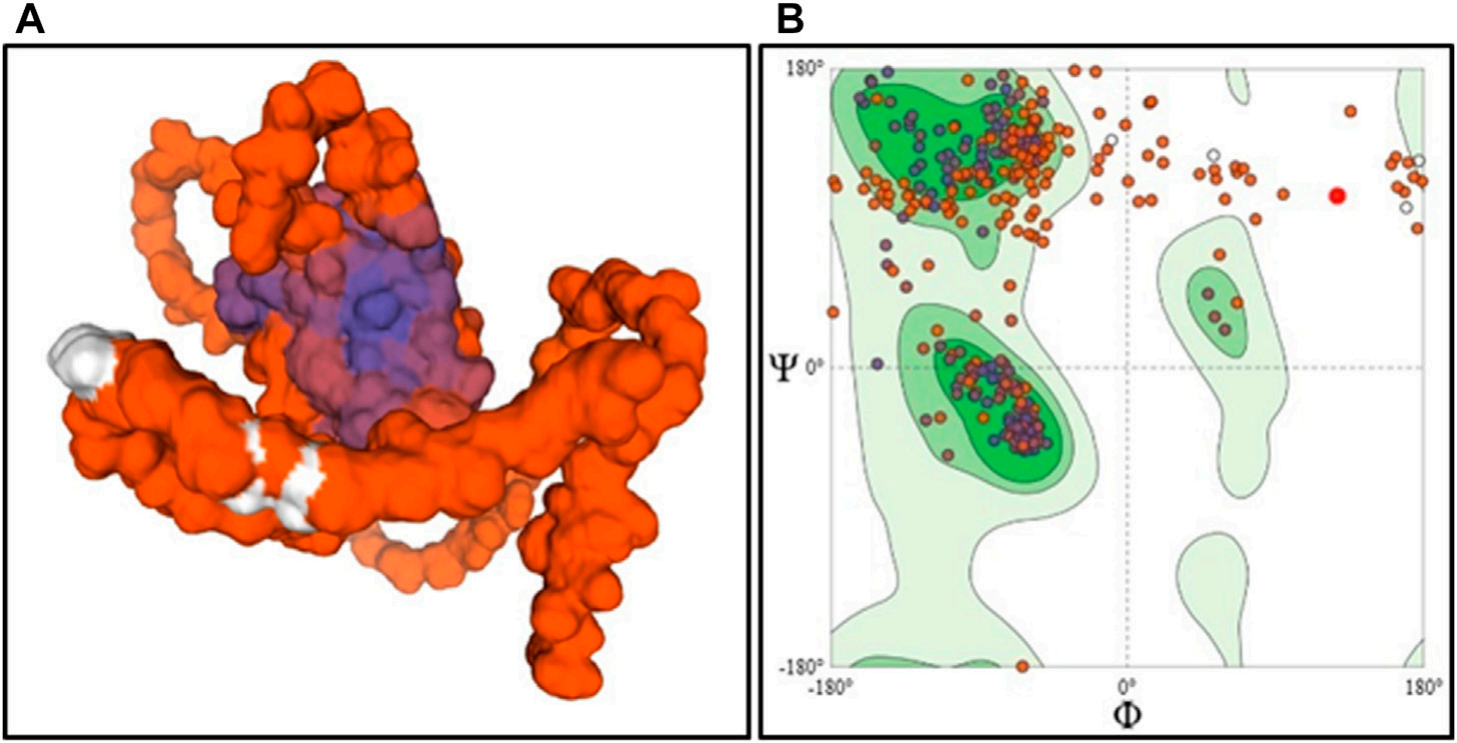
Homology model surface structure of ASTL determined by AlphaFold2 **(A)** and its Ramachandran Plot **(B)**.

The stable 3D models of wild type ASTL built using AlphaFold2 and I-TASSER were validated to identify the most stable structure. On comparison of the structural assessment of the AlphaFold2 vs. I-TASSER model, the AlphaFold2 model was found to have a higher Ramachandran favored region (73.43% vs. 57.11%), the minimum value for bad bonds and angles, clash score, and lesser MolProbity score (Supplementary Table S6). Hence, the AlphaFold2 model was employed for further analysis (Figure 5).

### 3.6 Comparison of wild type and ASTL mutants

**FIGURE 6:**
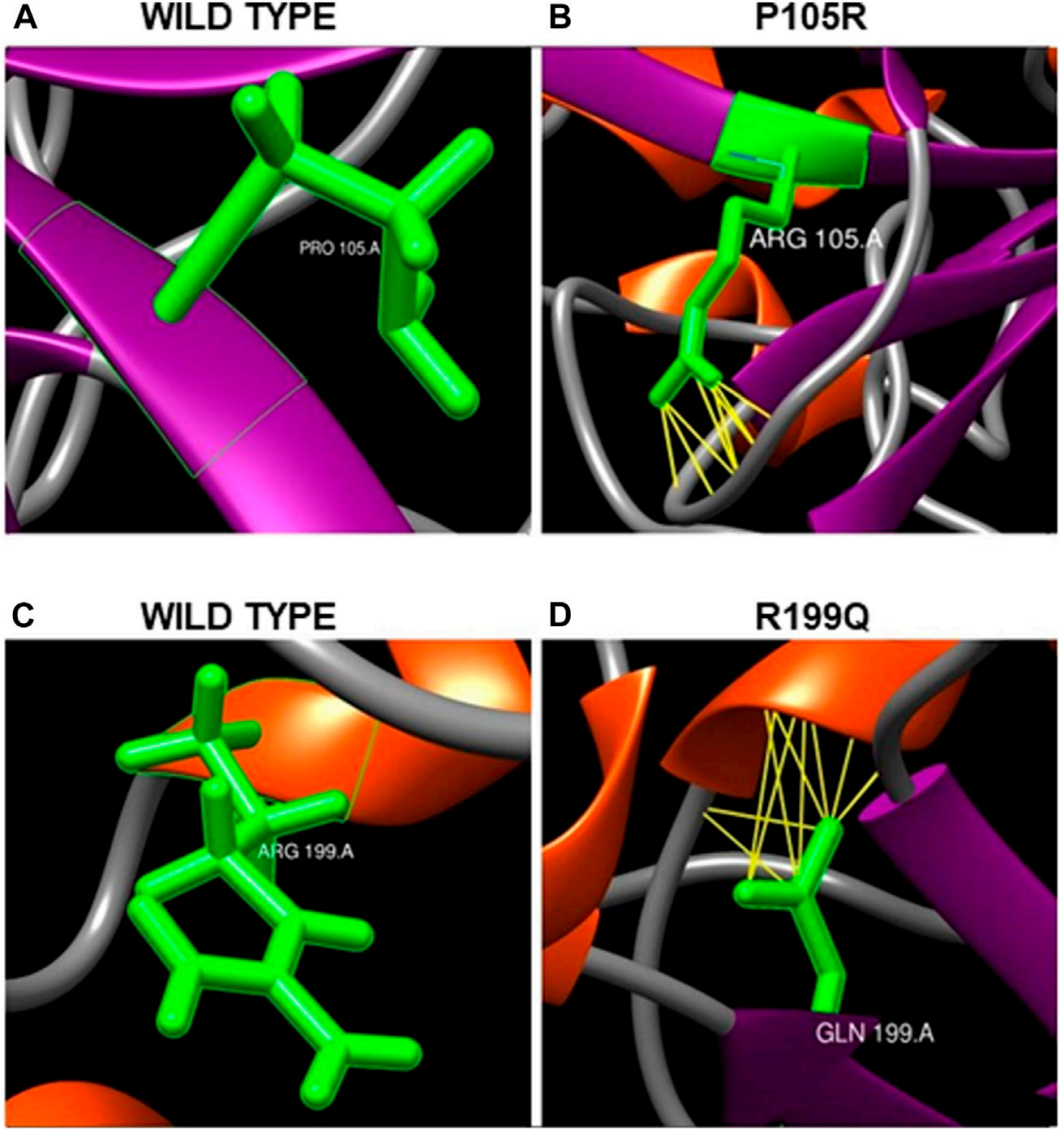
**(A,B)** Effect of single nucleotide variation rs150180534 (P105R) on ASTL’s 3D structure. **(A)** Proline at position 105 in wild type forms no contacts in native form whereas **(B)** Arginine at the same position forms thirty-four new contacts with Aspartic acid^143^, Arginine^142^ and Glutamine^141^. The networks of clashes or new pseudobonds are shown as yellow lines. **(C,D)** Effect of single nucleotide variation rs750874250 (R199Q) on protein’s 3D structure. **(C)** Arginine at position 199 position in wild type forms no contacts. **(D)** Glutamine at 199 position forms 15 contacts including pseudobonds with Histidine^194^ and Arginine^196^.

After minimization and optimization of the wild type and mutant ASTL structural models, they were utilized to examine the impact of these amino acid changes on the protein’s 3D structure and see whether any new networks of interactions or clashes had formed (Table 2). 35 out of the total of 40 nsSNPs resulted in novel networks of interactions or clashes. e.g., the substitution of P105R (Figure 6A) resulted in 34 new connections, including pseudobonds with Asp^143^, Arg^142^, and Gln^141^, which were absent in native protein (Figures 6A,B). Similarly, R199Q forms 15 new contacts including pseudobonds with His^194^ and Arg^196^ which were absent in the native protein (Figures 6C,D).

**TABLE 2:** List of 40 deleterious nsSNPs and the potential formation of a new network of collisions or contacts caused by the substituted amino acids at the relevant sites, as predicted by Chimera 1.16.

Substitution	New contacts	Amino acids with which pseudobonds formed
E75K	5	No
K93N	No	No
P105L	14	No
P105R	34	D^143^, R^142^, Q^141^
R117H	9	No
T131M	7	F^127^ and I^133^
F135S	1	No
G152R	41	I^63^, N^59^ and Q^60^
S155L	8	E^183^ and S^156^
R159H	6	No
G162E	0	No
H182L	3	No
H182R	13	No
L188P	9	L^184^ and M^185^
E193K	16	S^234^
R196W	16	N^225^ and E^193^
D198E	1	No
D198N	1	No
D198Y	5	No
R199Q	15	H^194^ and R^196^
R199W	13	R^196^
D200N	1	No
D200Y	10	No
R204C	2	No
E209K	0	No
I210F	13	No
Y230C	1	No
H237Y	26	D^231^
Y238C	1	No
Y238S	1	No
R246C	0	No
P249R	32	T^252^, P^253^and I^251^
P249L	12	R^240^ and I^251^
Q263R	12	S^233^
G262S	9	G^239^, Y^238^
R264Q	0	No
L267P	2	No
R274W	33	T^228^ and L^227^
L278P	7	R^274^
C281W	34	L^276^ and C^132^

Furthermore, TM-align and RMSD values were also calculated (Supplementary Table S7). The highest RMSD, predicted for rs145409944 (F135S) and rs780360443 (D198E), suggests that these SNPs have the highest deviation from the wild type model. The lowest value for TM-align, predicted for rs748161965 (E209K), points out that this SNP had the least similarity with the wild type model.

### 3.7 Molecular docking studies suggest hydrophobic interactions and H-bonding between ASTL and ZP2 and between ASTL and FETUB

Docking studies between the catalytic domain of ASTL and the ZP2 N-term region as well as the catalytic domain of ASTL and FETUB predicted both H bonding and hydrophobic interactions between the two. Eight amino acids of ASTL are predicted to be hydrogen bonded with the ZP2-N terminal region while 27 amino acids of ASTL were predicted to form hydrophobic contacts (Table 3). Similarly, with FETUB, 16 amino acids of ASTL are predicted to be hydrogen bonded with FETUB and 14 have hydrophobic interactions with FETUB (Table 4).

**TABLE 3:** List of amino acids in ASTL which are predicted to be H-bonded or form hydrophobic contacts with residues of ZP2.

ASTL	H Bonded to ZP2	Residues predicted to form hydrophobic contacts			
Glu^75^	Asn^37^	Ile^74^	Gly^97^	Lys^220^	Val^409^
Thr^90^	His^129^	Leu^87^	Gly^98^	Ser^221^	Glyc^411^
Thr^90^	Gly^130^	Ser^88^	Pro^105^	Ser^223^	Pro^413^
Gly^98^	Asn^123^	Ala^89^	Arg^142^	Leu^276^	
Glu^103^	Arg^128^	Ser^91^	Asp^142^	Lys^277^	
Arg^159^	Arg^128^	Trp^94^	Trp^191^	Pro^406^	
Gln^222^	Thr^34^	Pro^95^	Asp^200^	Val^407^	
His^424^	Asn^17^	Met^96^	Ile^219^	Pro^408^	

**TABLE 4:** List of amino acids in ASTL which are predicted to be H-bonded or form hydrophobic contacts with residues of FETUB.

ASTL	FETUB	Residues predicted to form hydrophobic contacts		
Thr^90^	Thr^150^	Ser^88^	Leu^227^	Ser^282^
Ser^91^	Asp^153^	Trp^94^	Lys^277^	Pro^283^
Lys^93^	Tyr^148^	Pro^95^	Leu^278^	
Met^96^	Ser^156^	Gln^139^	Gly^280^	
Gly^97^	Lys^144^	Asp^198^		
Gly^98^	Lys^144^	Arg^201^		
Gly^98^	Asp^309^	Ser^224^		
Glu^103^	Lys^313^	Met^226^		
Arg^142^	Met^149^			
Arg^159^	Met^149^			
Arg^159^	Tyr^148^			
Asn^225^	Trp^199^			
Tyr^279^	Ser^197^			
Cys^281^	Gln^166^			
Ser^284^	Asp^161^			
Arg^287^	Ser^163^			

### 3.8 Five SNPs affect gene regulation

Only five of the 774 SNPs submitted to FuncPred from both the coding and non-coding regions were predicted to influence the regulatory region of ASTL (Figure 1). It was recognized that the intron variations rs6727578 and rs57477571 affected transcription factor binding sites. Along with those, rs1724121 (5’ UTR variant, 500B downstream variant), rs2969490 (5’UTR variant, intron variant), and rs57072789 (3’UTR variant, 2 KB upstream variant) were also predicted to have an impact on transcription factor binding sites. The miRanda and Sanger microRNA binding sites were predicted to be affected by an SNP with rsID rs57072789. Thus, these SNPs probably modulate the expression of the ASTL gene at the transcription or translation level either by affecting the transcription factor binding sites or the miRNA binding sites.

### 3.9 Statistical Coupling Analysis of ASTL suggests that the amino acid positions susceptible to SNP and nsSNP variation are evolutionarily coupled

**FIGURE 7:**
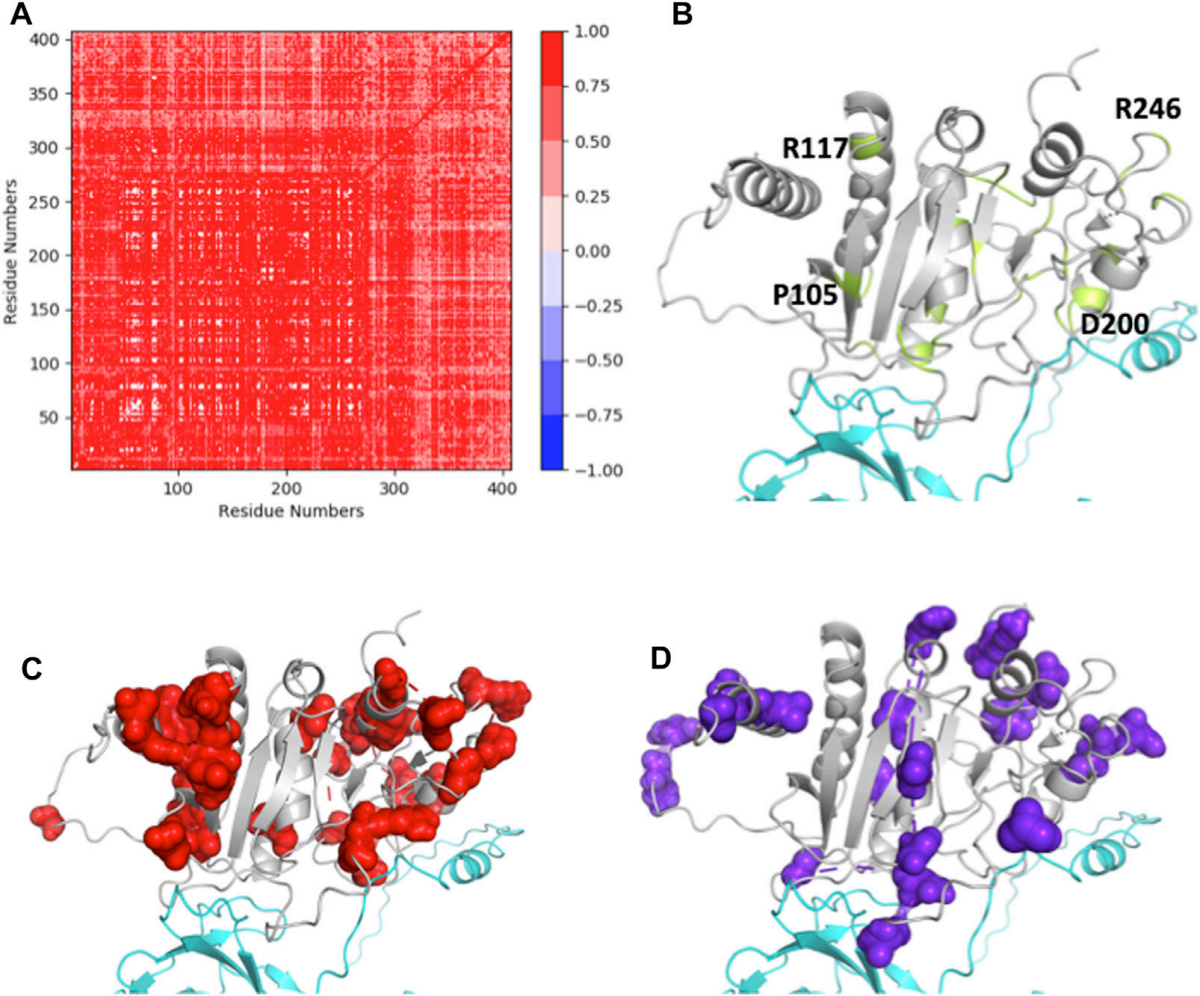
**(A)** SCA positional coevolution matrix. **(B)** Modeled structure of ASTL (grey), showing the putative ZP2 (cyan) interacting regions. The amino acid positions of ASTL susceptible to change are marked in green and few representative residues have been labeled **(C,D)** Two of the 8 independent sectors, IC1 and IC7, that fall in the ZP2 interacting region and also overlap with variable regions are shown in spheres.

The SCA performed using 471 ASTL sequences from the database, yielded 8 independent sectors (ICs), consisting of group of amino acid positions that are evolutionarily coupled in this family (Supplementary Figure S1). These positions have been mapped on the modeled structure of ASTL to understand their role in ASTL-ZP2 interaction. Clearly there is a considerable correlation between the ICs and the amino acid positions of ASTL that are prone to change on account of SNPs (Figure 7). Particularly, IC1 and IC7 could be mapped on the regions that interact with ZP2 and also overlap with the SNP dependent hyper-variable amino acid positions, suggesting that the mutations in ASTL could influence its interactions with ZP2.

## 4 Discussion

ASTL is predicted to influence female fertility as lack of ASTL activity post-fertilization or premature ASTL activity in unfertilized oocytes can prevent successful fertilization/embryo development. In light of the recent research associating a truncated variant of ASTL with infertility (Maddirevula et al., 2022), and the observation of altered fertility in *ASTL* ^−/−^ and *FETUB* ^−/−^ female mice (Burkart et al., 2012; Dietzel et al., 2013), we conducted an analysis of nsSNPs and regulatory SNPs in *ASTL* from the dbSNP database. Our objective was to gain a better understanding of their effect on ASTL activity and predict the SNPs which could impact the reproductive capability/capacity. Our study identified 40 disease-associated nsSNPs located in the catalytic domain of the protein (Figure 1). However, no clinical data as of now associates the shortlisted SNPs with female infertility in the EXOME/GWAS/WGS/ClinVar database. The low global MAF (<0.01) for these SNPs (except rs61735195), suggests that these SNPs are pathogenic (Supplementary Table S2). rs61735195 has a higher prevalence in the population as compared to other SNPs which may be because this SNP doesn’t lead to a structural or functional change in the protein as predicted by MutPred (Supplementary Table S3).

MAF values of these SNPs were unavailable for the Indian population in the ALPHA and ExAC databases. To address this gap, we consulted the Indigenomes database and out of the 40 nsSNPs, only rs61735195 was found (MAF = 0.0063) and had a higher prevalence just like it was observed for the global population (Figure 3). The absence of other rsIDs in the Indian population may be attributed to the limited sample size of the Indigenomes database, which has only sequenced over 1,000 whole genome sequences from India. This may be particularly true for SNPs with low MAF values where a larger sample size would be required to detect their presence.

Currently, only 12 nsSNPS of ASTL are listed in the Indigenomes database. Interestingly, one out of these is a unique nsSNP for the Indian population which leads to a G178D change (rs1273696164; MAF = 0.0005). This variant is predicted to be pathogenic by all the 5 *in silico* tools, with SNAP2 suggesting it to be an effect variant (Table 5). Furthermore, the ConSurf analysis indicates that the G^178^ residue is a highly conserved functional site (color grade 8). I-Mutant predicts a decrease in the stability of the mutant protein, with the TM-align and RMSD values indicating a structural difference from the wild type. MutPred2 suggests a gain of catalytic and allosteric sites at E^183^ and H^182^, respectively, and altered metal binding (Supplementary Table S3). It worth mentioning that E^183^ is the active site residue and H^182^ is one of the zinc binding residue for ASTL. Structural analysis using Chimera 1.16 predicted the formation of 20 new contacts including pseudobonds with R^264^ and W^265^.

**TABLE 5:** Evaluation of G178D (rs1273696164) by various *in silico* tools Evaluation of the pathogenicity of G178D.

Substitution	SIFT	PANTHER	POLYPHEN-2	SNPs&GO	PhD-SNP	SNAP2
G178D	-	Probably Damaging	Probably Damaging	Disease	Disease	Effect
		0.85	1.0	8	9	80
Structural and functional alterations in ASTL caused due to G178D as predicted by MutPred2						

**Substitution**	**MutPred 2 score**	**Alterations**	
G178D	0.872	Gain of Catalytic site at E^183^, Gain of Allosteric site at H^182^, Altered Metal binding, Altered Transmembrane protein	
Stability prediction for G178D using I-Mutant3.0			

**Substitution**	**Stability**	**Reliability index**	**Free energy change (Kcal/mol)**
G178D	Decrease	5	−0.78
TM-align and RMSD values of the mutant protein model			

**Substitution**	**TM score**	**RMSD values**
G178D	0.885	3.89

**FIGURE 8:**
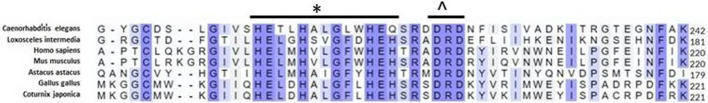
Sequence alignment of the ASTL protein. Conservation of ^198^DRD^200^ motif (represented as **^**) amongst various species. Upstream to DRD, the characteristic ^182^HExxHxxGxxHE^193^ motif (represented as *) of astacin metalloprotease is observed. A number of SNPs were found at the DRD.

Out of the 40 nsSNPs shortlisted in this study, 32 are present in the highly conserved regions as indicated by their high position-specific color grade (8–9; Supplementary Table S4; Figure 4). Seven of these positions have more than 1 SNP associated with them (2 SNPs at P^105^, H^182^, R^199^, D^200^, Y^238^, P^249^ and 3 SNPs at D^198^). Additionally, a concentration of SNPs was observed in the region around amino acids 193–210, which result in the formation of new contacts and or pseudobonds. This could be affecting the structure of the metalloprotease ASTL and/its interaction with its substrate, ZP2. In fact, the motif ^198^DRD^200^ which carries multiple SNPs at each position is highly conserved among different species highlighting its importance (Figure 8). Furthermore, SCA showed that the evolutionarily coupled and hence functionally important set of residues of ASTL overlapped with most of the amino acid positions in this protein that were susceptible to changes on account of SNPs. Nevertheless, experimental validation is required to confirm these observations.

As the crystal structure of human ASTL is yet to be determined, a homology model was built using AlphaFold2. Docking studies were then conducted to understand the type of interactions and amino acid residues involved in the interactions between ASTL and its substrate, ZP2. Both hydrophobic interactions (which may lead to alteration in the folding of the protein and subsequently decrease the stability of the protein) and H bonds were predicted between the two proteins (Tables 3, 4). The presence of SNPs at positions P^105^ and D^200^ (involved in hydrophobic interactions; and also present in the highly conserved region) and at E^75^ and R^159^ (positions associated with H bonding) further suggest that these positions are important. Lack of FETUB binding to ASTL can also cause infertility, hence docking studies of ASTL with its inhibitor were performed to see if any of the identified ASTL SNPs were affecting the binding of FETUB and hence fertility. SNPs were found at positions K^93^, R^159,^ and C^281^ of ASTL which were predicted to form H bond with FETUB; and at D^198^ and L^278^ which were predicted to have hydrophobic contacts. Multiple SNPs have been observed at positions P^105^, D^198^, and D^200^ of ASTL. Notably, the mutant D198E was observed to have the highest structural deviation from the WT in TM-align. Based on the studies by Cuppari et al., and our docking results, we looked for SNPs in FETUB and found a number of them to be present at positions associated with interaction with either crayfish astacin or human ASTL (e.g., F^110^, Y^148^, M^149^ T^150^, C^151^, P^152^, S^163^, Q^166^ and W^199^, V^200^ and V^201^; Unpublished results).

Post fertilization or in a chemically-induced egg activation, the mammalian egg releases zinc in a calcium-coordinated event termed the ‘zinc spark’ (Que et al., 2017). This zinc exocytosis modifies ZP which contributes to its function in preventing polyspermy. Human ASTL is a known metalloprotease that requires zinc for its activity. It contains a well-conserved 12-amino acid signature sequence ^182^HExxHxxGxxHE^193^ in which E^183^ acts as the active site residue by polarizing a water molecule implicated in a nucleophilic attack at the scissile peptide bond. The three histidine residues in the motif have been annotated as binding sites with the zinc ion. (Bode et al., 1992; Fukasawa et al., 2011). We also identified 2 SNPs, rs147042054 (H182L) and rs147042054 (H182R) at position 182 which possibly affect zinc binding and subsequently affect the activity of the protein.

The observations made in this study, are thus important as they predict the role of several amino acid residues in structure/activity/binding, the information that is currently lacking in the absence of a crystal structure for human ASTL. However, obtaining the crystal structure of human ASTL will strenghten our results and enable us to better understand the interaction of human ASTL with human ZP2 and FETUB. Experimental validation of the effect of SNPs on the structure/catalytic activity of ASTL using mutants is also necessary. Additionally, the structural data can be utilized to discover specific small-molecule inhibitors of ASTL that may be useful in the treatment of infertility in cases FETUB is non-functional or, in reducing failures in ART techniques like *in vitro* fertilization. It is also feasible that multiple SNPs, either alone or in combination with others, may have an impact on the structure/activity of the protein affecting fertility at the population level. To clinically validate the relationship between ASTL and infertility, whole genome sequencing, particularly of Idiopathic infertile females, may be more suitable. It is important to mention here that since ASTL is found to be highly expressed in brain and lymphoid tissue as compared to ovaries (https://www.proteinatlas.org/ENSG00000188886-ASTL/tissue#expression_summary), affected females may also show other phenotypes/co-morbidities along with infertility. The aforementioned studies will assist in elucidating the pathogenesis of unexplained infertility in females and in their identification and characterization, which may enhance their application in the diagnosis of female infertility, treatment and even in developing efficient contraceptives.

## Data Availability

The datasets presented in this study can be found in online repositories. The names of the repository/repositories and accession number(s) can be found below: dbSNP accession number: NM_001002036.4.
