# Supplementary figures and images for: In silico identification and characterization of the SNPs in the human ASTL gene and their probable role in female infertility
Source: Front Cell Dev Biol. 2023 Jun 8;11:1151672. doi: 10.3389/fcell.2023.1151672 (PMC10285486; doi:10.3389/fcell.2023.1151672)

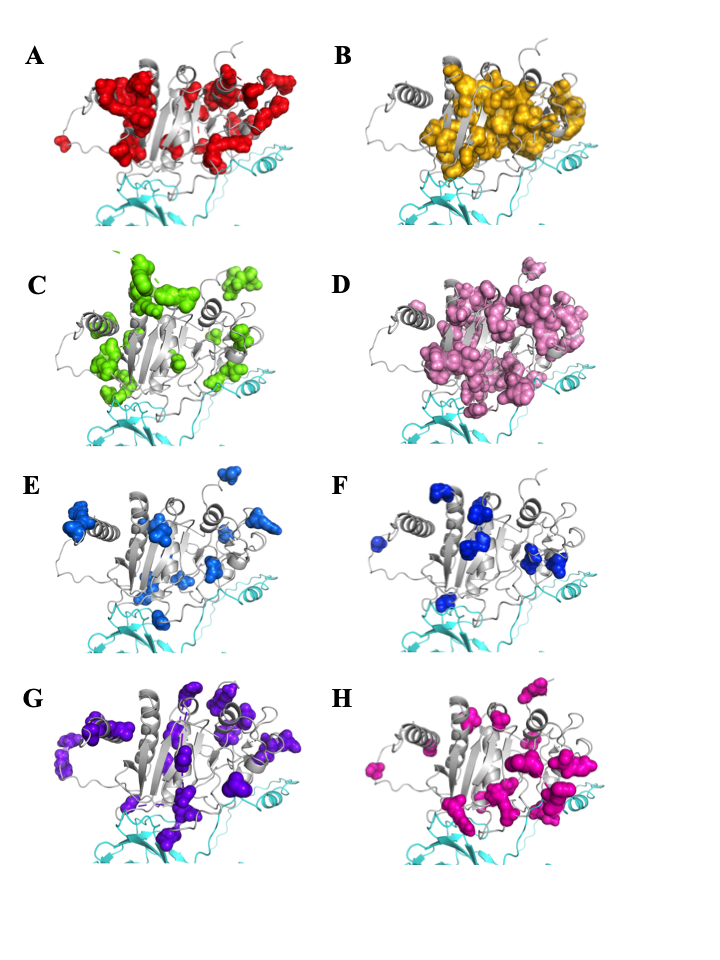

Supplement: Supplementary file 1 [file Image1.TIFF]
