# Supplementary material for: In silico identification and characterization of the SNPs in the human ASTL gene and their probable role in female infertility
Source: Front Cell Dev Biol. 2023 Jun 8;11:1151672. doi: 10.3389/fcell.2023.1151672 (PMC10285486; doi:10.3389/fcell.2023.1151672)
Supplement: Supplementary file 2 [file DataSheet1.docx]

# Table S1: Results of the 202 nsSNPs found in the ASTL gene using various bioinformatics tools. Numbers represent score or probability for each. Each nsSNP was first evaluated using SIFT, PANTHER, POLYPHEN-2, SNPs&GO and PhD-SNP. Only those nsSNPs which were predicted to be potentially deleterious by at least 4 out of the 5 tools were further evaluated by SNAP2. Green color represents benign/neutral. Red color represents potentially deleterious SNPs.

| **S. No.** | **Variant ID** | **Substitution** | **SIFT** | **PANTHER** | **POLYPHEN-2** | **SNPs&GO** | **PhD-SNP** | **SNAP2** |
| --- | --- | --- | --- | --- | --- | --- | --- | --- |
| 1. | rs777889105 | G3D | Deleterious | - | Benign | Neutral | Neutral |  |
|  |  |  | 0 |  | 0.038 | 5 | 8 |  |
| 2. | rs747531394 | G3S | Deleterious | - | Benign | Neutral | Neutral |  |
|  |  |  | 0 |  | 0 | 8 | 8 |  |
| 3. | rs777889105 | G3V | Deleterious | - | Benign | Neutral | Neutral |  |
|  |  |  | 0 |  | 0.038 | 6 | 4 |  |
| 4. | rs748327712 | L7P | Deleterious | - | Probably Damaging | Neutral | Neutral |  |
|  |  |  | 0 |  | 0.999 | 3 | 1 |  |
| 5. | rs748327712 | L7R | Deleterious | - | Probably Damaging | Neutral | Disease | Effect |
|  |  |  | 0 |  | 0.999 | 3 | 0 | 71 |
| 6. | rs780915411 | L17M | Deleterious | - | Probably Damaging | Neutral | Neutral |  |
|  |  |  | 0 |  | 0.982 | 8 | 6 |  |
| 7. | rs757203100 | P18S | Tolerated | - | Benign | Neutral | Neutral |  |
|  |  |  | 0.08 |  | 0.041 | 7 | 7 |  |
| 8. | rs757203100 | P18T | Tolerated | - | Benign | Neutral | Neutral |  |
|  |  |  | 0.08 |  | 0.001 | 9 | 7 |  |
| 9. | rs751499117 | G19S | Deleterious | - | Probably damaging | Neutral | Neutral |  |
|  |  |  | 0.04 |  | 0.964 | 6 | 7 |  |
| 10. | rs757915689 | L22P | Deleterious | - | Probably damaging | Neutral | Neutral |  |
|  |  |  | 0 |  | 0.971 | 5 | 4 |  |
| 11. | rs752251902 | A24P | Deleterious | - | Possibly Damaging | Neutral | Neutral |  |
|  |  |  | 0.02 |  | 0.799 | 5 | 6 |  |
| 12. | rs569867697 | A24V | Deleterious | - | Benign | Neutral | Neutral |  |
|  |  |  | 0.02 |  | 0.001 | 9 | 9 |  |
| 13. | rs753476623 | A27D | Deleterious | - | Possibly Damaging | Neutral | Neutral |  |
|  |  |  | 0 |  | 0.906 | 4 | 0 |  |
| 14. | rs766729086 | S29I | Tolerated | - | Benign | Neutral | Neutral |  |
|  |  |  | 0.07 |  | 0.697 | 5 | 6 |  |
| 15. | rs749493910 | F38L | Tolerated | - | Benign | Neutral | Neutral |  |
|  |  |  | 0.91 |  | 0 | 9 | 6 |  |
| 16. | rs775599202 | P39S | Tolerated | - | Benign | Neutral | Neutral |  |
|  |  |  | 0.1 |  | 0 | 9 | 9 |  |
| 17. | rs746785099 | L42R | Deleterious | - | Probably Damaging | Neutral | Neutral |  |
|  |  |  | 0.02 |  | 0.971 | 5 | 2 |  |
| 18. | rs141044848 | T43I | Deleterious | - | Benign | Neutral | Neutral | Neutral |
|  |  |  | 0 |  | 0.002 | 8 | 7 |  |
| 19. | rs754481406 | T47N | Deleterious | - | Possibly Damaging | Neutral | Neutral |  |
|  |  |  | 0.02 |  | 0.906 | 8 | 6 |  |
| 20. | rs754481406 | T47S | Tolerated | - | Benign | Neutral | Neutral |  |
|  |  |  | 0.82 |  | 0.036 | 7 | 9 |  |
| 21. | rs756432623 | S50C | Tolerated | - | Probably Damaging | Neutral | Neutral |  |
|  |  |  | 0.09 |  | 0.983 | 7 | 7 |  |
| 22. | rs756432623 | S50F | Tolerated | - | Benign | Neutral | Neutral |  |
|  |  |  | 0.21 |  | 0.005 | 8 | 6 |  |
| 23. | rs767926152 | G51R | Tolerated | - | Benign | Neutral | Neutral |  |
|  |  |  | 0.26 |  | 0 | 6 | 5 |  |
| 24. | rs752100184 | D52N | Deleterious | - | Probably Damaging | Neutral | Neutral |  |
|  |  |  | 0.03 |  | 1 | 7 | 1 |  |
| 25. | rs764193295 | K53N | Deleterious | - | Possibly | Neutral | Neutral |  |
|  |  |  | 0 |  | Damaging | 8 | 3 |  |
|  |  |  |  |  | 0.718 |  |  |  |
| 26. | rs775715292 | P56L | Tolerated | - | Probably Damaging | Neutral | Neutral |  |
|  |  |  | 0.12 |  | 1 | 8 | 6 |  |
| 27. | rs762956336 | P56S | Tolerated | - | Probably Damaging | Neutral | Neutral |  |
|  |  |  | 0.21 |  | 1 | 9 | 9 |  |
| 28. | rs769905379 | A57V | Tolerated | - | Possibly | Neutral | Neutral |  |
|  |  |  | 0.08 |  | Damaging | 8 | 6 |  |
|  |  |  |  |  | 0.761 |  |  |  |
| 29. | rs759861243 | I58V | Deleterious | - | Probably Damaging | Neutral | Neutral |  |
|  |  |  | 0.05 |  | 0.984 | 8 | 5 |  |
| 30. | rs370555704 | S70N | Tolerated | - | Benign | Neutral | Neutral |  |
|  |  |  | 0.1 |  | 0.057 | 9 | 8 |  |
| 31. | rs532123683 | S71T | Tolerated | - | Possibly | Neutral | Neutral |  |
|  |  |  | 0.12 |  | Damaging | 9 | 9 |  |
|  |  |  |  |  | 0.759 |  |  |  |
| 32. | rs753291024 | I74M | Tolerated | - | Possibly | Neutral | Neutral |  |
|  |  |  | 0.06 |  | Damaging | 9 | 6 |  |
|  |  |  |  |  | 0.742 |  |  |  |
| 33. | rs771568000 | E75D | Tolerated | - | Probably Damaging | Neutral | Disease |  |
|  |  |  | 0.07 |  | 0.999 | 5 | 1 |  |
| 34. | rs765193573 | E75K | Deleterious | - | Probably Damaging | Disease | Disease | Effect |
|  |  |  | 0.01 |  | 1 | 1 | 8 |  |
| 35. | rs745520994 | I78V | Deleterious | - | Probably Damaging | Neutral | Neutral |  |
|  |  |  | 0.04 |  | 0.984 | 8 | 1 |  |
| 36. | rs184519754 | I79F | Tolerated | - | Benign | Neutral | Disease |  |
|  |  |  | 0.12 |  | 0.392 | 6 | 1 |  |
| 37. | rs774001295 | I79T | Tolerated | - | Benign | Neutral | Neutral |  |
|  |  |  | 0.05 |  | 0.155 | 8 | 3 |  |
| 38. | rs184519754 | I79V | Tolerated | - | Benign | Neutral | Neutral |  |
|  |  |  | 0.19 |  | 0.001 | 9 | 8 |  |
| 39. | rs373382016 | R80Q | Tolerated | - | Probably Damaging | Neutral | Neutral |  |
|  |  |  | 0.14 |  | 0.997 | 5 | 5 |  |
| 40. | rs377535345 | R80W | Tolerated | - | Probably Damaging | Neutral | Neutral |  |
|  |  |  | 0.12 |  | 0.96 | 4 | 6 |  |
| 41. | rs775529258 | P81L | Tolerated | - | Probably Damaging | Disease | Neutral |  |
|  |  |  | 0.06 |  | 0.998 | 2 | 2 |  |
| 42. | rs755524379 | K93N | Deleterious | - | Probably Damaging | Disease | Disease | Effect |
|  |  |  | 0.03 |  | 0.986 | 1 | 3 | 29 |
| 43. | rs749857645 | S99G | Tolerated | - | Benign | Neutral | Neutral |  |
|  |  |  | 0.99 |  | 0 | 8 | 6 |  |
| 44. | rs781489619 | V102L | Deleterious | - | Probably Damaging | Neutral | Disease |  |
|  |  |  | 0.04 |  | 1 | 8 | 1 |  |
| 45. | rs781489619 | V102M | Deleterious | - | Probably Damaging | Neutral | Neutral |  |
|  |  |  | 0.01 |  | 1 | 8 | 0 |  |
| 46. | rs150180534 | P105L | Deleterious | Probably Damaging | Probably Damaging | Disease | Disease | Effect |
|  |  |  | 0.02 | 0.85 | 1 | 5 | 7 | 80 |
| 47. | rs150180534 | P105R | Deleterious | Probably Damaging | Probably Damaging | Disease | Disease | Effect |
|  |  |  | 0.02 | 0.85 | 1 | 6 | 6 | 86 |
| 48. | rs765068047 | L108F | Tolerated | - | Possibly | Neutral | Neutral |  |
|  |  |  | 0.24 |  | Damaging | 6 | 2 |  |
|  |  |  |  |  | 0.871 |  |  |  |
| 49. | rs753806992 | S109Y | Deleterious | - | Probably Damaging | Neutral | Neutral |  |
|  |  |  | 0.03 |  | 0.999 | 0 | 2 |  |
|  |  |  |  |  |  |  |  |  |
| 50. | rs760249581 | D113N | Tolerated | - | Benign | Neutral | Neutral |  |
|  |  |  | 0.15 |  | 0.031 | 6 | 4 |  |
| 51. | rs774176117 | R117H | Deleterious | Probably Damaging | Probably Damaging | Neutral | Disease | Effect |
|  |  |  | 0.01 | 0.74 | 1 | 1 | 2 | 62 |
| 52. | rs763422497 | I120V | Tolerated | - | Probably Damaging | Neutral | Neutral |  |
|  |  |  | 0.19 |  | 0.773 | 8 | 1 |  |
| 53. | rs376411675 | L124F | Tolerated | - | Benign | Neutral | Disease |  |
|  |  |  | 0.27 |  | 0 | 9 | 1 |  |
| 54. | rs376411675 | L124V | Tolerated | - | Benign | Neutral | Neutral |  |
|  |  |  | 0.17 |  | 0.112 | 8 | 0 |  |
| 55. | rs201102363 | R129H | Deleterious | - | Benign | Neutral | Disease |  |
|  |  |  | 0.05 |  | 0.003 | 1 | 0 |  |
| 56. | rs201102363 | R129L | Tolerated | - | Benign | Disease | Disease |  |
|  |  |  | 0.27 |  | 0.321 | 3 | 5 |  |
| 57. | rs747187625 | S130F | Tolerated | - | Benign | Neutral | Neutral |  |
|  |  |  | 0.71 |  | 0 | 9 | 0 |  |
| 58. | rs777987842 | T131M | Deleterious | Probably Damaging | Probably Damaging | Disease | Disease | Effect |
|  |  |  | 0.05 | 0.85 | 1 | 1 | 9 | 67 |
| 59. | rs145409944 | F135S | Deleterious | Probably Damaging | Probably Damaging | Disease | Disease | Effect |
|  |  |  | 0.01 | 0.85 | 0.999 | 7 | 8 | 82 |
| 60. | rs756052392 | I148F | Tolerated | - | Possibly | Neutral | Disease |  |
|  |  |  | 0.87 |  | Damaging | 6 | 1 |  |
|  |  |  |  |  | 0.71 |  |  |  |
| 61. | rs201203679 | M150I | Tolerated | - | Possibly Damaging | Neutral | Disease |  |
|  |  |  | 0.46 |  | 0.454 | 4 | 1 |  |
| 62. | rs767011047 | M150T | Tolerated | - | Probably Damaging | Neutral | Neutral |  |
|  |  |  | 0.61 |  | 0.891 | 1 | 2 |  |
| 63. | rs762760157 | G152R | Deleterious | Probably Damaging | Probably Damaging | Disease | Disease | Effect |
|  |  |  | 0.01 | 0.85 | 1 | 7 | 8 | 85 |
| 64. | rs759833663 | S155L | Deleterious | Probably Damaging | Probably Damaging | Disease | Disease | Effect |
|  |  |  | 0 | 0.85 | 1 | 5 | 7 | 87 |
| 65. | rs766795734 | S156R | Tolerated | - | Probably Damaging | Disease | Disease |  |
|  |  |  | 0.4 |  | 0.863 | 2 | 6 |  |
| 66. | rs61735195 | R159H | Deleterious | Probably Damaging | Benign | Disease | Disease | Effect |
|  |  |  | 0.02 | 0.78 | 0.952 | 2 | 6 | 55 |
| 67. | rs774455538 | G162E | Tolerated | Probably Damaging | Probably Damaging | Disease | Disease | Effect |
|  |  |  | 0.1 | 0.57 | 1 | 4 | 7 | 79 |
| 68. | rs769572571 | V166A | Deleterious | - | Possibly | Neutral | Neutral |  |
|  |  |  | 0 |  | Damaging | 3 | 3 |  |
|  |  |  |  |  | 0.757 |  |  |  |
| 69. | rs777378437 | P170A | Tolerated | - | Probably Damaging | Neutral | Neutral |  |
|  |  |  | 0.43 |  | 0.994 | 6 | 7 |  |
| 70. | rs200103398 | T171M | Tolerated | - | Probably Damaging | Neutral | Neutral |  |
|  |  |  | 0.11 |  | 0.894 | 2 | 4 |  |
| 71. | rs766783182 | R177Q | Tolerated | - | Benign | Neutral | Neutral |  |
|  |  |  | 0.33 |  | 0.016 | 7 | 4 |  |
| 72. | rs754143947 | R177W | Tolerated | - | Probably Damaging | Neutral | Disease |  |
|  |  |  | 0.19 |  | 0.99 | 5 | 5 |  |
| 73. | rs761136005 | I179V | Tolerated | Probably Damaging | Probably Damaging | Neutral | Neutral |  |
|  |  |  | 0.29 | 0.85 | 0.775 | 8 | 7 |  |
| 74. | rs767533400 | L181I | Tolerated | - | Probably Damaging | Neutral | Neutral |  |
|  |  |  | 0.37 |  | 0.993 | 8 | 8 |  |
| 75. | rs147042054 | H182L | Deleterious | Probably Damaging | Probably Damaging | Disease | Disease | Effect |
|  |  |  | 0 | 0.85 | 1 | 8 | 9 | 86 |
| 76. | rs147042054 | H182R | Deleterious | Probably Damaging | Probably Damaging | Disease | Disease | Effect |
|  |  |  | 0.01 | 0.85 | 1 | 8 | 9 | 88 |
| 77. | rs139152188 | V187A | Tolerated | Probably Damaging | Benign | Neutral | Neutral |  |
|  |  |  | 0.74 | 0.85 | 0.659 | 5 | 6 |  |
| 78. | rs768755515 | L188P | Deleterious | - | Probably Damaging | Disease | Disease | Effect |
|  |  |  | 0 |  | 1 | 7 | 8 | 86 |
| 79. | rs746895908 | E193K | Deleterious | Probably Damaging | Probably Damaging | Disease | Disease | Effect |
|  |  |  | 0.01 | 0.85 | 1 | 7 | 9 | 89 |
| 80. | rs371727585 | T195A | Tolerated | Probably Damaging | Benign | Neutral | Neutral |  |
|  |  |  | 0.21 | 0.85 | 0 | 7 | 2 |  |
| 81. | rs368781391 | R196W | Deleterious | Probably Damaging | Probably Damaging | Disease | Disease | Effect |
|  |  |  | 0 | 0.85 | 1 | 8 | 9 | 84 |
| 82. | rs780360443 | D198E | Deleterious | Probably Damaging | Probably Damaging | Disease | Disease | Effect |
|  |  |  | 0.02 | 0.85 | 0.999 | 5 | 5 | 56 |
| 83. | rs753572086 | D198N | Deleterious | Probably Damaging | Probably Damaging | Disease | Disease | Effect |
|  |  |  | 0 | 0.85 | 1 | 5 | 7 | 65 |
| 84. | rs753572086 | D198Y | Deleterious | Probably Damaging | Probably Damaging | Disease | Disease | Effect |
|  |  |  | 0 | 0.85 | 1 | 8 | 7 | 76 |
| 85. | rs750874250 | R199Q | Deleterious | Probably Damaging | Probably Damaging | Disease | Disease | Effect |
|  |  |  | 0.02 | 0.85 | 1 | 7 | 8 | 85 |
| 86. | rs151115044 | R199W | Deleterious | Probably Damaging | Probably Damaging | Disease | Disease | Effect |
|  |  |  | 0 | 0.85 | 1 | 8 | 7 | 92 |
| 87. | rs751579283 | D200N | Deleterious | Probably Damaging | Probably Damaging | Disease | Disease | Effect |
|  |  |  | 0.02 | 0.85 | 0.896 | 5 | 6 | 48 |
| 88. | rs751579283 | D200Y | Deleterious | Probably Damaging | Probably Damaging | Disease |  | Effect |
|  |  |  | 0 | 0.85 | 1 | 8 |  | 72 |
| 89. | rs775876067 | R201H | Deleterious | - | Benign | Disease | Disease |  |
|  |  |  | 0.04 |  | 0.06 | 1 | 1 |  |
| 90. | rs143172084 | R204C | Deleterious | - | Probably | Disease | Disease |  |
|  |  |  | 0.04 |  | Damaging | 3 | 5 |  |
|  |  |  |  |  | 0.995 |  |  |  |
| 91. | rs143172084 | R204G | Tolerated | - | Possibly | Neutral | Disease |  |
|  |  |  | 0.32 |  | Damaging | 3 | 5 |  |
|  |  |  |  |  | 0.546 |  |  |  |
| 92. | rs41320144 | R204H | Deleterious | - | Benign | Neutral | Neutral |  |
|  |  |  | 0.04 |  | 0.019 | 5 | 1 |  |
| 93. | rs143172084 | R204S | Tolerated | - | Possibly Damaging | Neutral | Neutral |  |
|  |  |  | 0.83 |  | 0.546 | 5 | 1 |  |
| 94. | rs772118993 | N208K | Tolerated | - | Benign | Disease | Neutral |  |
|  |  |  | 0.18 |  | 0.065 | 0 | 1 |  |
| 95. | rs748161965 | E209K | Tolerated | - | Probably Damaging | Disease | Disease |  |
|  |  |  | 0.31 |  | 0.999 | 4 | 6 |  |
| 96. | rs748161965 | E209Q | Tolerated | - | Probably Damaging | Neutral | Disease |  |
|  |  |  | 0.24 |  | 0.999 | 2 | 1 |  |
| 97. | rs201337537 | I210F | Deleterious | Probably Damaging | Probably Damaging | Disease | Disease | Effect |
|  |  |  | 0 | 0.78 | 0.992 | 8 | 9 | 47 |
| 98. | rs201337537 | I210V | Tolerated | Probably Damaging | Benign | Neutral | Neutral |  |
|  |  |  | 0.22 | 0.78 | 0.181 | 3 | 4 |  |
| 99. | rs758892466 | I216V | Tolerated | - | Benign | Neutral | Neutral |  |
|  |  |  | 0.5 |  | 0.126 | 9 | 8 |  |
| 100. | rs752809649 | I219F | Tolerated | - | Possibly Damaging | Neutral | Disease |  |
|  |  |  | 0.09 |  | 0.71 | 3 | 3 |  |
| 101. | rs752809649 | I219V | Tolerated | - | Benign | Neutral | Neutral |  |
|  |  |  | 0.31 |  | 0.001 | 9 | 9 |  |
| 102. | rs754140470 | S223N | Tolerated | - | Benign | Neutral | Neutral |  |
|  |  |  | 0.27 |  | 0.01 | 3 | 1 |  |
| 103. | rs373134495 | Q222K | Tolerated | - | Benign | Neutral | Neutral |  |
|  |  |  | 0.7 |  | 0.004 | 8 | 3 |  |
| 104. | rs749458 | Q222L | Tolerated | - | Benign | Neutral | Neutral |  |
|  |  |  | 0.23 |  | 0.02 | 6 | 4 |  |
| 105. | rs749458 | Q222P | Tolerated | - | Benign | Neutral | Disease |  |
|  |  |  | 0.21 |  | 0 | 0 | 5 |  |
| 106. | rs749458 | Q222R | Tolerated | - | Benign | Neutral | Disease |  |
|  |  |  | 0.83 |  | 0 | 9 | 0 |  |
| 107. | rs767172858 | T228M | Tolerated | - | Probably Damaging | Neutral | Neutral |  |
|  |  |  | 0.16 |  | 0.965 | 8 | 4 |  |
| 108. | rs774093278 | Y230C | Deleterious | Probably Damaging | Probably Damaging | Disease | Disease | Effect |
|  |  |  | 0 | 0.85 | 1 | 7 | 7 | 87 |
| 109 | rs201932296 | H237Y | Deleterious | Probably Damaging | Probably Damaging | Disease | Disease | Effect |
|  |  |  | 0 | 0.85 | 1 | 2 | 8 | 70 |
| 110 | rs762938662 | Y238C | Tolerated | Probably Damaging | Probably Damaging | Disease | Disease | Effect |
|  |  |  | 0 | 0.85 | 1 | 7 | 9 | 79 |
| 111 | rs762938662 | Y238S | Deleterious | Probably Damaging | Probably Damaging | Disease | Disease | Effect |
|  |  |  | 0 | 0.85 | 1 | 7 | 9 | 91 |
| 112. | rs548158177 | A242T | Tolerated | - | Probably Damaging | Neutral | Disease |  |
|  |  |  | 0.19 |  | 1 | 4 | 5 |  |
| 113. | rs748612996 | R245Q | Tolerated | Probably Damaging | Possibly | Neutral | Neutral |  |
|  |  |  | 0.66 | 0.74 | Damaging | 6 | 2 |  |
|  |  |  |  |  | 0.888 |  |  |  |
| 114. | rs139129570 | R245W | Tolerated | Probably Damaging | Benign | Neutral | Disease |  |
|  |  |  | 0.27 | 0.74 | 0.028 | 8 | 7 |  |
| 115. | rs543411711 | R246C | Deleterious | - | Probably Damaging | Disease | Disease | Effect |
|  |  |  | 0.04 |  | 1 | 6 | 4 | 11 |
| 116. | rs749378316 | R246H | Tolerated | - | Probably Damaging | Disease | Disease |  |
|  |  |  | 0.1 |  | 0.99 | 2 | 2 |  |
| 117. | rs780058774 | P249L | Tolerated | - | Probably Damaging | Disease | Disease | Effect |
|  |  |  | 0.03 |  | 0.992 | 1 | 5 | 18 |
| 118. | rs780058774 | P249R | Deleterious | - | Probably Damaging | Disease | Disease | Effect |
|  |  |  | 0.05 |  | 0.999 | 1 | 7 | 25 |
| 119. | rs756339024 | T252I | Tolerated | - | Benign | Neutral | Disease |  |
|  |  |  | 0.64 |  | 0.001 | 9 | 1 |  |
| 120. | rs756339024 | T252K | Tolerated | - | Possibly Damaging | Neutral | Disease |  |
|  |  |  | 0.39 |  | 0.586 | 4 | 3 |  |
| 121. | rs777585652 | A256V | Tolerated | - | Benign | Neutral | Neutral |  |
|  |  |  | 0.29 |  | 0.44 | 7 | 2 |  |
| 122. | rs758211826 | S258G | Tolerated | - | Benign | Neutral | Neutral |  |
|  |  |  | 0.15 |  | 0.001 | 5 | 8 |  |
| 123. | rs752564077 | S258N | Tolerated | - | Benign | Neutral | Neutral |  |
|  |  |  | 0.29 |  | 0.002 | 2 | 9 |  |
| 124. | rs753364550 | G262S | Deleterious | Probably Damaging | Probably Damaging | Disease | Disease | Effect |
|  |  |  | 0 | 0.85 | 1 | 7 | 8 | 66 |
| 125. | rs765844957 | Q263R | Deleterious | Probably Damaging | Probably Damaging | Disease | Disease | Effect |
|  |  |  | 0.03 | 0.85 | 0.997 | 1 | 8 | 10 |
| 126. | rs772758672 | R264Q | Deleterious | Probably Damaging | Probably Damaging | Disease | Disease | Effect |
|  |  |  | 0.01 | 0.85 | 1 | 2 | 5 | 63 |
| 127. | rs72935897 | N266K | Tolerated | - | Probably Damaging | Neutral | Neutral |  |
|  |  |  | 0.53 |  | 0.975 | 2 | 4 |  |
| 128. | rs774925080 | L267P | Tolerated | Probably Damaging | Probably Damaging | Disease | Disease | Effect |
|  |  |  | 0.08 | 0.85 | 1 | 7 | 7 | 69 |
| 129. | rs769275452 | S270L | Tolerated | - | Benign | Disease | Neutral |  |
|  |  |  | 0.65 |  | 0.027 | 0 | 7 |  |
| 130. | rs551825040 | R274Q | Tolerated | - | Probably Damaging | Neutral | Neutral |  |
|  |  |  | 0.18 |  | 0.999 | 3 | 7 |  |
| 131. | rs568621983 | R274W | Deleterious | - | Probably Damaging | Disease | Disease | Effect |
|  |  |  | 0 |  |  | 6 | 7 | 75 |
| 132. | rs371568317 | V275L | Tolerated | - | Probably Damaging | Neutral | Neutral |  |
|  |  |  | 0.34 |  | 0.99 | 6 | 9 |  |
| 133. | rs1657502 | K277E | Tolerated | Probably Damaging | Benign | Neutral | Disease |  |
|  |  |  | 0.11 | 0.78 | 0.224 | 5 | 1 |  |
| 134. | rs371081763 | K277T | Tolerated | Probably Damaging | Possibly Damaging | Neutral | Neutral |  |
|  |  |  | 0.2 | 0.78 | 0.586 | 8 | 5 |  |
| 135. | rs747922771 | L278P | Deleterious | Probably Damaging | Possibly Damaging | Disease | Disease | Effect |
|  |  |  | 0.01 | 0.85 | 1 | 6 | 8 | 83 |
| 136. | rs765861589 | C281W | Deleterious | Probably Damaging | Probably Damaging | Disease | Disease | Effect |
|  |  |  | 0 | 0.85 | 1 | 8 | 7 | 85 |
| 137. | rs755588781 | P283Q | Tolerated | - | Benign | Neutral | Neutral |  |
|  |  |  | 0.43 |  | 0.021 | 8 | 8 |  |
| 138. | rs764595372 | G290E | Deleterious | - | Possibly Damaging | Neutral | Neutral |  |
|  |  |  | 0.05 |  | 0.906 | 9 | 7 |  |
| 139. | rs149434198 | R291K | Tolerated | - | Benign | Neutral | Neutral |  |
|  |  |  | 0.13 |  | 0.004 | 9 | 8 |  |
| 140. | rs764505450 | S293F | Tolerated | - | Benign | Neutral | Neutral |  |
|  |  |  | 0.93 |  | 0 | 10 | 6 |  |
| 141. | rs753172521 | G299R | Deleterious | - | Benign | Neutral | Neutral |  |
|  |  |  | 0.01 |  | 0.392 | 8 | 7 |  |
| 142. | rs565777679 | P304L | Deleterious | - | Benign | Neutral | Neutral |  |
|  |  |  | 0 |  | 0.027 | 8 | 4 |  |
| 143. | rs369203898 | A305T | Deleterious | - | Benign | Neutral | Neutral |  |
|  |  |  | 0.01 |  | 0.2 | 9 | 8 |  |
| 144. | rs749035810 | L309M | Deleterious | - | Probably Damaging | Neutral | Neutral |  |
|  |  |  | 0.02 |  | 1 | 9 | 8 |  |
| 145. | rs751116126 | R311Q | Deleterious | - | Benign | Neutral | Neutral |  |
|  |  |  | 0.01 |  | 0 | 8 | 8 |  |
| 146. | rs143968684 | R311W | Deleterious | - | Possibly Damaging | Neutral | Neutral |  |
|  |  |  | 0 |  | 0.916 | 6 | 7 |  |
| 147. | rs780810636 | A315V | Tolerated | - | Benign | Neutral | Neutral |  |
|  |  |  | 0.07 |  | 0.414 | 9 | 9 |  |
| 148. | rs371728783 | S317L | Deleterious | - | Benign | Neutral | Neutral |  |
|  |  |  | 0 |  | 0.001 | 9 | 9 |  |
| 149. | rs144613777 | A318E | Tolerated | - | Benign | Neutral | Neutral |  |
|  |  |  | 1 |  | 0.003 | 9 | 9 |  |
| 150. | rs144613777 | A318V | Tolerated | - | Benign | Neutral | Neutral |  |
|  |  |  | 0.1 |  | 0.002 | 9 | 9 |  |
| 151. | rs754361962 | E319G | Deleterious | - | Benign | Neutral | Neutral |  |
|  |  |  | 0 |  | 0.005 | 9 | 8 |  |
| 152. | rs147267731 | E319K | Deleterious | - | Benign | Neutral | Neutral |  |
|  |  |  | 0 |  | 0.437 | 8 | 8 |  |
| 153. | rs760745469 | D324N | Tolerated | - | Possibly Damaging | Neutral | Neutral |  |
|  |  |  | 0.35 |  | 0.804 | 10 | 10 |  |
| 154. | rs773350568 | P325A | Deleterious | - | Benign | Neutral | Neutral |  |
|  |  |  | 0 |  | 0 | 10 | 9 |  |
| 155. | rs773350568 | P325T | Deleterious | - | Benign | Neutral | Neutral |  |
|  |  |  | 0 |  | 0.041 | 10 | 9 |  |
| 156. | rs570403802 | A330V | Tolerated | - | Possibly Damaging | Neutral | Neutral |  |
|  |  |  | 0.05 |  | 0.814 | 9 | 7 |  |
| 157. | rs547207590 | Q333R | Deleterious | - | Benign | Neutral | Neutral |  |
|  |  |  | 0 |  | 0.001 | 9 | 8 |  |
| 158. | rs770500068 | P334L | Deleterious | - | Benign | Neutral | Neutral |  |
|  |  |  | 0.01 |  | 0.072 | 10 | 9 |  |
| 159. | rs199510888 | V335I | Tolerated | - | Benign | Neutral | Neutral |  |
|  |  |  | 1 |  | 0 | 10 | 8 |  |
| 160. | rs757954964 | E341K | Tolerated | - | Benign | Neutral | Neutral |  |
|  |  |  | 0.18 |  | 0.041 | 8 | 7 |  |
| 161. | rs779094841 | H344R | Tolerated | - | Benign | Neutral | Neutral |  |
|  |  |  | 0.1 |  | 0 | 10 | 9 |  |
| 162. | rs755346666 | W346R | Deleterious | - | Benign | Neutral | Neutral |  |
|  |  |  | 0 |  | 0.001 | 7 | 4 |  |
| 163. | rs754272008 | E347K | Deleterious | - | Benign | Neutral | Neutral |  |
|  |  |  | 0 |  | 0.002 | 9 | 9 |  |
| 164. | rs754272008 | E347Q | Deleterious | - | Benign | Neutral | Neutral |  |
|  |  |  | 0 |  | 0.062 | 9 | 7 |  |
| 165. | rs766863005 | S348A | Tolerated | - | Benign | Neutral | Neutral |  |
|  |  |  | 0.6 |  | 0.118 | 9 | 9 |  |
| 166. | rs756675234 | S348F | Tolerated | - | Benign | Neutral | Neutral |  |
|  |  |  | 0.07 |  | 0.004 | 8 | 7 |  |
| 167. | rs766863005 | S348P | Tolerated | - | Benign | Neutral | Neutral |  |
|  |  |  | 0.29 |  | 0 | 10 | 9 |  |
| 168. | rs766863005 | S348T | Tolerated | - | Benign | Neutral | Neutral |  |
|  |  |  | 0.71 |  | 0.118 | 9 | 9 |  |
| 169. | rs750451671 | K352R | Tolerated | - | Benign | Neutral | Neutral |  |
|  |  |  | 0.18 |  | 0 | 10 | 9 |  |
| 170. | rs761903802 | E357G | Tolerated | - | Benign | Neutral | Neutral |  |
|  |  |  | 0.53 |  | 0.002 | 9 | 9 |  |
| 171. | rs767679190 | E357Q | Tolerated | - | Possibly Damaging | Neutral | Neutral |  |
|  |  |  | 0.29 |  | 0.845 | 8 | 8 |  |
| 172. | rs774657969 | S359L | Tolerated | - | Benign | Neutral | Neutral |  |
|  |  |  | 1 |  | 0.005 | 9 | 8 |  |
| 173. | rs776635886 | T365S | Tolerated | - | Benign | Neutral | Neutral |  |
|  |  |  | 0.25 |  | 0.004 | 9 | 7 |  |
| 174. | rs746981708 | P370L | Deleterious | - | Benign | Neutral | Neutral |  |
|  |  |  | 0.01 |  | 0 | 9 | 9 |  |
| 175. | rs746981708 | P370Q | Deleterious | - | Benign | Neutral | Neutral |  |
|  |  |  | 0.01 |  | 0.001 | 9 | 8 |  |
| 176. | rs772556539 | R371K | Tolerated | - | Benign | Neutral | Neutral |  |
|  |  |  | 1 |  | 0.002 | 10 | 9 |  |
| 177. | rs772556539 | R371T | Deleterious | - | Benign | Neutral | Neutral |  |
|  |  |  | 0.01 |  | 0.267 | 8 | 9 |  |
| 178. | rs771633909 | S372T | Deleterious | - | Possibly | Neutral | Neutral |  |
|  |  |  | 0 |  | Damaging | 9 | 7 |  |
|  |  |  |  |  | 0.759 |  |  |  |
| 179. | rs778629357 | P374T | Deleterious | - | Probably Damaging | Neutral | Neutral |  |
|  |  |  | 0 |  | 0.975 | 8 | 8 |  |
| 180. | rs754689379 | G375A | Deleterious | - | Benign | Neutral | Neutral |  |
|  |  |  | 0 |  | 0.001 | 8 | 8 |  |
| 181. | rs749642739 | A376V | Deleterious | - | Benign | Neutral | Neutral |  |
|  |  |  | 0 |  | 0.331 | 8 | 8 |  |
| 182. | rs756587078 | A378D | Deleterious | - | Possibly | Neutral | Neutral |  |
|  |  |  | 0 |  | Damaging | 7 | 5 |  |
|  |  |  |  |  | 0.838 |  |  |  |
| 183. | rs559739374 | A378S | Deleterious | - | Possibly Damaging | Neutral | Neutral |  |
|  |  |  | 0 |  | 0.596 | 8 | 7 |  |
| 184. | rs756587078 | A378V | Deleterious | - | Benign | Neutral | Neutral |  |
|  |  |  | 0 |  | 0.002 | 9 | 9 |  |
| 185. | rs767448744 | P379L | Tolerated | - | Possibly | Neutral | Neutral |  |
|  |  |  | 0.07 |  | Damaging | 8 | 9 |  |
|  |  |  |  |  | 0.845 |  |  |  |
| 186. | rs750974865 | P379S | Tolerated | - | Possibly | Neutral | Neutral |  |
|  |  |  | 0.26 |  | Damaging | 8 | 8 |  |
|  |  |  |  |  | 0.608 |  |  |  |
| 187. | rs137865237 | G380D | Deleterious | - | Benign | Neutral | Neutral |  |
|  |  |  | 0.03 |  | 0.011 | 3 | 8 |  |
| 188. | rs200446698 | G380S | Tolerated | - | Benign | Neutral | Neutral |  |
|  |  |  | 0.99 |  | 0.213 | 10 | 9 |  |
| 189. | rs776533698 | V381D | Deleterious | - | Benign | Neutral | Neutral |  |
|  |  |  | 0 |  | 0.012 | 4 | 5 |  |
| 190. | rs200898499 | Q383R | Tolerated | - | Benign | Neutral | Neutral |  |
|  |  |  | 0.07 |  | 0.137 | 7 | 9 |  |
| 191. | rs757814790 | G390R | Tolerated | - | Benign | Neutral | Neutral |  |
|  |  |  | 0.15 |  | 0.017 | 5 | 8 |  |
| 192. | rs771412793 | A402G | Deleterious | - | Benign | Neutral | Neutral |  |
|  |  |  | 0 |  | 0.02 | 8 | 8 |  |
| 193. | rs747687030 | G403R | Tolerated | - | Benign | Neutral | Neutral |  |
|  |  |  | 0.9 |  | 0.02 | 9 | 9 |  |
| 194. | rs773788833 | I404S | Tolerated | - | Benign | Neutral | Neutral |  |
|  |  |  | 0.43 |  | 0 | 7 | 4 |  |
| 195. | rs372982717 | S412N | Deleterious | - | Benign | Neutral | Neutral |  |
|  |  |  | 0.01 |  | 0 | 10 | 8 |  |
| 196. | rs770169651 | A414D | Tolerated | - | Probably Damaging | Neutral | Disease |  |
|  |  |  | 0.06 |  | 0.982 | 6 | 1 |  |
| 197. | rs781724839 | P416L | Deleterious | - | Possibly Damaging | Neutral | Neutral |  |
|  |  |  | 0 |  | 0.59 | 4 | 3 |  |
| 198. | rs746302992 | P416S | Deleterious | - | Possibly | Neutral | Neutral |  |
|  |  |  | 0 |  | Damaging | 4 | 3 |  |
|  |  |  |  |  | 0.59 |  |  |  |
| 199. | rs746302992 | P416T | Deleterious | - | Probably Damaging | Neutral | Neutral |  |
|  |  |  | 0 |  | 0.982 | 4 | 2 |  |
| 200. | rs577986927 | N423H | Deleterious | - | Benign | Neutral | Neutral |  |
|  |  |  | 0.02 |  | 0.214 | 7 | 9 |  |
| 201. | rs760519375 | E430D | Tolerated | - | Benign | Neutral | Neutral |  |
|  |  |  | 0.09 |  | 0.003 | 9 | 9 |  |
| 202. | rs200161060 | E430K | Tolerated | - | Benign | Neutral | Neutral |  |
|  |  |  | 0.21 |  | 0 | 8 | 9 |  |

SIFT score: Deleterious ≤ 0.05 and Tolerated > 0.05; *Panther score shows the damaging probability (PDel) values of the SNPs;* PolyPhen-2 score: probably damaging = 0.950-1, possibly damaging = 0.850-0.950, benign= 0; PhD-SNP probability value: disease ≥0.50, neutral <0.50; SNPs&GO probability value: disease ≥0.50, neutral <0.50; *SNAP 2 score shows the effect, score and expected accuracy percentage of the SNPs.*

# Table S2: Minor allele frequency of the 40 shortlisted SNPs in a) ExAc and b) Alfa database. Table S2b includes only those rsIDs whose MAF data was unavailable in ExAc.

**a)**

| Minor Allele Frequency (ExAC) | | | | | | | | |
| --- | --- | --- | --- | --- | --- | --- | --- | --- |
| **S. No** | **rsID** | **Substitution** | **Total** | **European** | **Asian** | **American** | **African** | **Other** |
| 1 | rs765193573 | E75K | 0.000008 | 0.00001 | 0.00000 | 0.00000 | 0.00000 | 0.000 |
| 2 | rs755524379 | K93N | 0.000008 | 0.00001 | 0.00000 | 0.00000 | 0.00000 | 0.000 |
| 4 | rs150180534 | P105R | 0.000008 | 0.00001 | 0.00000 | 0.00000 | 0.00010 | 0.000 |
| 5 | rs774176117 | R117H | 0.000017 | 0.00001 | 0.00004 | 0.00000 | 0.00000 | 0.000 |
| 6 | rs777987842 | T131M | 0.000008 | 0.00001 | 0.00000 | 0.00000 | 0.00000 | 0.000 |
| 7 | rs145409944 | F135S | 0.000041 | 0.00007 | 0.00000 | 0.00000 | 0.00000 | 0.000 |
| 8 | rs762760157 | G152R | 0.000008 | 0.00001 | 0.00000 | 0.00000 | 0.00000 | 0.000 |
| 9 | rs759833663 | S155L | 0.000008 | 0.00001 | 0.00000 | 0.00000 | 0.00000 | 0.000 |
| 10 | rs61735195 | R159H | 0.012433 | 0.01781 | 0.00396 | 0.00598 | 0.00205 | 0.015 |
| 11 | rs774455538 | G162E | 0.000008 | 0.00000 | 0.00000 | 0.00009 | 0.00000 | 0.000 |
| 12 | rs147042054 | H182R | 0.000041 | 0.00007 | 0.00000 | 0.00000 | 0.00000 | 0.000 |
| 13 | rs768755515 | L188P | 0.000008 | 0.00001 | 0.00000 | 0.00000 | 0.00000 | 0.000 |
| 14 | rs746895908 | E193K | 0.000017 | 0.00003 | 0.00000 | 0.00000 | 0.00000 | 0.000 |
| 15 | rs368781391 | R196W | 0.000058 | 0.00004 | 0.00008 | 0.00009 | 0.00000 | 0.001 |
| 16 | rs780360443 | D198E | 0.000008 | 0.00000 | 0.00004 | 0.00000 | 0.00000 | 0.000 |
| 17 | rs753572086 | D198Y | 0.000008 | 0.00000 | 0.00000 | 0.00000 | 0.00000 | 0.000 |
| 18 | rs750874250 | R199Q | 0.000008 | 0.00000 | 0.00004 | 0.00000 | 0.00000 | 0.000 |
| 19 | rs151115044 | R199W | 0.000075 | 0.00006 | 0.00012 | 0.00009 | 0.0001 | 0.000 |
| 20 | rs751579283 | D200N | 0.000025 | 0.00000 | 0.00012 | 0.00000 | 0.00000 | 0.000 |
| 21 | rs774093278 | Y230C | 0.000008 | 0.00001 | 0.00000 | 0.00000 | 0.00000 | 0.000 |
| 22 | rs762938662 | Y238S | 0.000008 | 0.00001 | 0.00000 | 0.00000 | 0.00000 | 0.000 |
| 23 | rs543411711 | R246C | 0.000025 | 0.00003 | 0.00004 | 0.00000 | 0.00000 | 0.000 |
| 24 | rs780058774 | P249R | 0.000008 | 0.00001 | 0.00004 | 0.00000 | 0.00000 | 0.000 |
| 25 | rs753364550 | G262S | 0.000025 | 0.00000 | 0.00000 | 0.00000 | 0.00029 | 0.000 |
| 26 | rs765844957 | Q263R | 0.000008 | 0.00001 | 0.00000 | 0.00000 | 0.00000 | 0.000 |
| 27 | rs772758672 | R264Q | 0.000008 | 0.00000 | 0.00000 | 0.00000 | 0.00010 | 0.000 |
| 28 | rs774925080 | L267P | 0.000008 | 0.00000 | 0.00004 | 0.00000 | 0.00000 | 0.000 |
| 29 | rs568621983 | R274W | 0.000025 | 0.00003 | 0.00004 | 0.00000 | 0.00000 | 0.000 |
| 30 | rs747922771 | L278P | 0.000009 | 0.00000 | 0.00005 | 0.00000 | 0.00000 | 0.000 |
| 31 | rs765861589 | C281W | 0.000054 | 0.00006 | 0.00000 | 0.00000 | 0.0002 | 0.000 |

| **Minor Allele Frequency (ALFA)** | | | | | | | |
| --- | --- | --- | --- | --- | --- | --- | --- |
| **S. No** | **rsID** | **Substitution** | **Total** | **European** | **Asian** | **African** | **Other** |
| 1 | rs753572086 | D198N | 0.00009 | 0.0001 | 0.000 | 0.0000 | 0.000 |
| 2 | rs143172084 | R204C | 0.003957 | 0.004279 | 0.000 | 0.0004 | 0.00356 |
| 3 | rs748161965 | E209K | 0.00000 | 0.0000 | 0.000 | 0.0000 | 0.000 |
| 4 | rs201932296 | H237Y | 0.00000 | 0.0000 | 0.000 | 0.0000 | 0.000 |
| 5 | rs762938662 | Y238C | 0.00000 | 0.0000 | 0.000 | 0.0000 | 0.000 |
| 6 | rs780058774 | P249L | 0.00000 | 0.0000 | 0.000 | 0.0000 | 0.000 |

# Table S3: Structural and functional alterations in ASTL caused by the shortlisted deleterious nsSNPs as predicted by MutPred2.

| **S.No** | **Variant ID** | **Substitution** | **MutPred 2 score** | **Alterations** |
| --- | --- | --- | --- | --- |
| 1 | rs765193573 | E75K | 0.910 | Altered Metal binding, Altered Transmembrane protein, Gain of Strand, Loss of Catalytic site at D^77^, Altered Stability |
| 2 | rs755524379 | K93N | 0.393 | No alterations observed |
| 3 | rs150180534 | P105L | 0.743 | Altered Transmembrane protein, Gain of Allosteric site at F^106^, Altered Metal binding |
| 4 | rs150180534 | P105R | 0.831 | Altered Transmembrane protein, Altered Ordered interface, Gain of Strand, Gain of Allosteric site at F^106^, Altered Metal binding, Gain of Proteolytic cleavage at P^105^ |
| 5 | rs774176117 | R117H | 0.115 | No alterations observed |
| 6 | rs777987842 | T131M | 0.549 | Altered Transmembrane protein, Altered Ordered interface, Loss of Relative solvent accessibility, Loss of Allosteric site at F^135^, Altered Metal binding, Gain of Disulfide linkage at C^132^, Loss of Catalytic site at C^132^ |
| 7 | rs145409944 | F135S | 0.891 | Gain of Relative solvent accessibility, Altered Ordered interface, Altered Stability, Altered Transmembrane protein, Loss of Allosteric site at F^135^, Altered Metal binding, Gain of Disulfide linkage at C^132^, Gain of Catalytic site at C^132^ |
| 8 | rs762760157 | G152R | 0.949 | Altered Ordered interface, Loss of Strand, Gain of Disulfide linkage at C^153^, Altered Transmembrane protein, Loss of Catalytic site at C^153^, Loss of Sulfation at Y^151^ |
| 9 | rs759833663 | S155L | 0.842 | Altered Ordered interface, Altered Metal binding, Loss of Strand, Loss of Disulfide linkage at C^153^, Altered Transmembrane protein, Loss of Catalytic site at C^153^, Loss of Sulfation at Y^151^ |
| 10 | rs61735195 | R159H | 0.296 | No alterations observed |
| 11 | rs774455538 | G162E | 0.82 | Altered Transmembrane protein, Altered Ordered interface, Loss of Strand, Gain of Pyrrolidone carboxylic acid at Q^164^ |
| 12 | rs147042054 | H182L | 0.963 | Altered Metal binding, Loss of Allosteric site at H^182^, Loss of Strand, Gain of Catalytic site at H^186^ |
| 13 | rs147042054 | H182R | 0.932 | Altered Metal binding, Loss of Catalytic site at E^183^, Gain of Allosteric site at H^182^, Gain of Helix, Loss of Strand |
| 14 | rs768755515 | L188P | 0.854 | Altered Metal binding, Gain of Catalytic site at E^183^, Loss of Helix, Gain of Allosteric site at E^183^ |
| 15 | rs746895908 | E193K | 0.847 | Loss of Catalytic site at E^193^, Altered Metal binding, Gain of Allosteric site at R^196^ |
| 16 | rs368781391 | R196W | 0.807 | Gain of Catalytic site at E^193^, Altered Ordered interface, Altered Metal binding, Loss of Relative solvent accessibility, Loss of Allosteric site at R^196^, Altered Transmembrane protein |
| 17 | rs780360443 | D198E | 0.817 | Altered Metal binding, Altered Ordered interface, Loss of Relative solvent accessibility, Gain of Allosteric site at R^196^, Altered Transmembrane protein, Gain of Catalytic site at E^193^ |
| 18 | rs753572086 | D198N | 0.875 | Altered Ordered interface, Altered Metal binding, Gain of Allosteric site at R^196^, Loss of Relative solvent accessibility, Altered Transmembrane protein, Loss of Catalytic site at E^193^ |
| 19 | rs753572086 | D198Y | 0.929 | Altered Ordered interface, Loss of Catalytic site at E^193^, Altered Metal binding, Gain of Allosteric site at R^196^, Loss of Relative solvent accessibility, Altered Transmembrane protein, Gain of Sulfation at D^198^ |
| 20 | rs750874250 | R199Q | 0.8 | Altered Metal binding, Altered Ordered interface, Gain of Strand, Loss of Relative solvent accessibility, Gain of Allosteric site at R^196^, Altered Transmembrane protein, Loss of Catalytic site at R^199^ |
| 21 | rs151115044 | R199W | 0.873 | Altered Ordered interface, Loss of Relative solvent accessibility, Altered Metal binding, Gain of Strand, Altered Disordered interface, Gain of Allosteric site at R^196^, Altered Transmembrane protein, Gain of Catalytic site at R^196^ |
| 22 | rs751579283 | D200N | 0.848 | Altered Metal binding, Altered Ordered interface, Altered Transmembrane protein, Gain of Allosteric site at R^196^, Loss of Relative solvent accessibility, Loss of Catalytic site at R^199^ |
| 23 | rs751579283 | D200Y | 0.924 | Altered Metal binding, Altered Disordered interface, Loss of Relative solvent accessibility, Altered Ordered interface, Gain of Allosteric site at R^196^, Altered Transmembrane protein, Loss of Catalytic site at R^199^ |
| 24 | rs143172084 | R204C | 0.378 | No alterations observed |
| 25 | rs748161965 | E209K | 0.735 | Altered Metal binding, Altered Transmembrane protein, Gain of Allosteric site at E^209^, Altered Disordered interface, Gain of Relative solvent accessibility, Loss of Loop |
| 26 | rs201337537 | I210F | 0.843 | Gain of Allosteric site at I^210^, Altered Disordered interface, Altered Metal binding, Gain of Loop, Loss of Relative solvent accessibility |
| 27 | rs774093278 | Y230C | 0.882 | Altered Metal binding, Altered Transmembrane protein, Altered Ordered interface, Loss of Relative solvent accessibility, Loss of Allosteric site at Y^232^, Loss of Catalytic site at D^231^, Gain of Disulfide linkage at Y^230^, Loss of Sulfation at Y^232^ |
| 28 | rs201932296 | H237Y | 0.84 | Altered Metal binding, Altered Ordered interface, Gain of Allosteric site at Y232, Altered Transmembrane protein, Loss of Catalytic site at Y^232^, Gain of Sulfation at Y^232^ |
| 29 | rs762938662 | Y238C | 0.915 | Altered Ordered interface, Altered Metal binding, Altered Transmembrane protein, Gain of Allosteric site at H^237^, Gain of Relative solvent accessibility, Loss of Catalytic site at Y^238^ |
| 30 | rs762938662 | Y238S | 0.917 | Altered Ordered interface, Altered Metal binding, Gain of Relative solvent accessibility, Altered Transmembrane protein, Loss of Allosteric site at Y^238^, Loss of Catalytic site at Y^238^ |
| 31 | rs543411711 | R246C | 0.597 | Loss of Relative solvent accessibility, Altered Transmembrane protein, Altered Ordered interface, Altered Metal binding, Loss of O-linked glycosylation at T^250^ |
| 32 | rs780058774 | P249L | 0.59 | Altered Ordered interface, Altered Transmembrane protein, Loss of Relative solvent accessibility, Altered Metal binding, Loss of O-linked glycosylation at T^250^ |
| 33 | rs780058774 | P249R | 0.62 | Gain of Relative solvent accessibility, Altered Transmembrane protein, Altered Ordered interface, Altered Metal binding, Loss of O-linked glycosylation at T^250^ |
| 34 | rs753364550 | G262S | 0.901 | Altered Ordered interface, Altered Transmembrane protein, Altered DNA binding, Altered Metal binding, Loss of N-linked glycosylation at N^266^, Gain of Catalytic site at Q^263^ |
| 35 | rs765844957 | Q263R | 0.854 | Altered Disordered interface, Altered Transmembrane protein, Altered DNA binding, Altered Metal binding, Gain of Allosteric site at L^267^, Loss of N-linked glycosylation at N^266^, Loss of Catalytic site at Q^263^ |
| 36 | rs772758672 | R264Q | 0.738 | Altered Transmembrane protein, Altered Disordered interface, Altered DNA binding, Altered Metal binding, Gain of Catalytic site at Q^263^, Loss of N-linked glycosylation at N^266^ |
| 37 | rs774925080 | L267P | 0.912 | Altered Transmembrane protein, Altered Ordered interface, Altered Disordered interface, Altered DNA binding, Altered Metal binding, Loss of Allosteric site at L^267^, Loss of N-linked glycosylation at N^266^, Altered Stability, Loss of Catalytic site at Q^263^ |
| 38 | rs568621983 | R274W | 0.563 | Altered Ordered interface, Altered Transmembrane protein |
| 39 | rs747922771 | L278P | 0.876 | Gain of Intrinsic disorder, Loss of Disulfide linkage at C^281^, Altered Stability, Altered Transmembrane protein |
| 40 | rs765861589 | C281W | 0.917 | Altered Disordered interface, Altered Metal binding, Altered Ordered interface, Loss of Disulfide linkage at C^281^, Altered Transmembrane protein |

# Table S4: Stability prediction of the shortlisted 40nsSNPs using I-mutant 3.0

| **S.No** | **Variant ID** | **Substitution** | **Stability** | **Reliability Index** | **Free energy Change (Kcal/mol)** |
| --- | --- | --- | --- | --- | --- |
| 1 | rs765193573 | E75K | Decrease | 6 | -0.61 |
| 2 | rs755524379 | K93N | Decrease | 1 | -0.62 |
| 3 | rs150180534 | P105L | Decrease | 4 | -0.67 |
| 4 | rs150180534 | P105R | Decrease | 6 | -1.04 |
| 5 | rs774176117 | R117H | Decrease | 9 | -1.4 |
| 6 | rs777987842 | T131M | Decrease | 1 | -0.02 |
| 7 | rs145409944 | F135S | Decrease | 8 | -1.94 |
| 8 | rs762760157 | G152R | Decrease | 7 | -0.68 |
| 9 | rs759833663 | S155L | Decrease | 0 | -0.04 |
| 10 | rs61735195 | R159H | Decrease | 8 | -1.32 |
| 11 | rs774455538 | G162E | Increase | 3 | -0.47 |
| 12 | rs147042054 | H182L | Increase | 3 | 0.56 |
| 13 | rs147042054 | H182R | Decrease | 1 | 0.12 |
| 14 | rs768755515 | L188P | Decrease | 6 | -1.39 |
| 15 | rs746895908 | E193K | Decrease | 8 | -0.9 |
| 16 | rs368781391 | R196W | Decrease | 6 | -0.5 |
| 17 | rs780360443 | D198E | Increase | 1 | -0.36 |
| 18 | rs753572086 | D198N | Decrease | 2 | -0.79 |
| 19 | rs753572086 | D198Y | Increase | 0 | -0.05 |
| 20 | rs750874250 | R199Q | Decrease | 9 | -1.25 |
| 21 | rs151115044 | R199W | Decrease | 6 | -0.59 |
| 22 | rs751579283 | D200N | Decrease | 1 | -0.64 |
| 23 | rs751579283 | D200Y | Increase | 2 | 0.03 |
| 24 | rs143172084 | R204C | Decrease | 7 | -1.26 |
| 25 | rs748161965 | E209K | Decrease | 8 | -0.86 |
| 26 | rs201337537 | I210F | Decrease | 9 | -1.51 |
| 27 | rs774093278 | Y230C | Decrease | 3 | -1.17 |
| 28 | rs201932296 | H237Y | Decrease | 6 | -0.11 |
| 29 | rs762938662 | Y238C | Decrease | 5 | -1.51 |
| 30 | rs762938662 | Y238S | Decrease | 8 | -1.77 |
| 31 | rs543411711 | R246C | Decrease | 5 | -1.06 |
| 32 | rs780058774 | P249L | Decrease | 6 | -0.6 |
| 33 | rs780058774 | P249R | Decrease | 8 | -1.04 |
| 34 | rs753364550 | G262S | Decrease | 9 | -1.22 |
| 35 | rs765844957 | Q263R | Increase | 2 | -0.13 |
| 36 | rs772758672 | R264Q | Decrease | 8 | -1.05 |
| 37 | rs774925080 | L267P | Decrease | 5 | -1.72 |
| 38 | rs568621983 | R274W | Decrease | 5 | -0.52 |
| 39 | rs747922771 | L278P | Decrease | 5 | -1.73 |
| 40 | rs765861589 | C281W | Decrease | 2 | -0.25 |

# Table S5: Conservation score and Color grade prediction for the 40 nsSNPs of ASTL

| **S. No** | **Variant ID** | **Substitutions** | **Conservation score** | **Color grade** | **Position of residues** |
| --- | --- | --- | --- | --- | --- |
| 1 | rs765193573 | E75K | -0.827 | 7 | Exposed |
| 2 | rs755524379 | K93N | 0.052 | 5 | Exposed |
| 4 | rs150180534 | P105L | -1.342 | 9 | Buried and structural residue |
| 3 | rs150180534 | P105R | -1.342 | 9 | Buried and structural residue |
| 5 | rs774176117 | R117H | -0.393 | 6 | Exposed |
| 6 | rs777987842 | T131M | -1.497 | 9 | Buried and structural residue |
| 7 | rs145409944 | F135S | -1.457 | 9 | Buried and structural residue |
| 8 | rs762760157 | G152R | -1.446 | 9 | Buried and structural residue |
| 9 | rs759833663 | S155L | -1.507 | 9 | Buried and functional residue |
| 10 | rs61735195 | R159H | -0.834 | 7 | Exposed |
| 11 | rs774455538 | G162E | -1.324 | 9 | Exposed and functional residue |
| 13 | rs147042054 | H182L | -1.552 | 9 | Buried and structural residue |
| 12 | rs147042054 | H182R | -1.552 | 9 | Buried and structural residue |
| 14 | rs768755515 | L188P | -0.907 | 8 | Buried |
| 15 | rs746895908 | E193K | -1.544 | 9 | Exposed and functional residue |
| 16 | rs368781391 | R196W | -1.513 | 9 | Exposed and functional residue |
| 17 | rs780360443 | D198E | -1.458 | 9 | Exposed and functional residue |
| 18 | rs753572086 | D198N | -1.458 | 9 | Exposed and functional residue |
| 19 | rs753572086 | D198Y | -1.458 | 9 | Exposed and functional residue |
| 20 | rs750874250 | R199Q | -1.548 | 9 | Exposed and functional residue |
| 21 | rs151115044 | R199W | -1.548 | 9 | Exposed and functional residue |
| 22 | rs751579283 | D200N | -1.513 | 9 | Exposed and functional residue |
| 23 | rs751579283 | D200Y | -1.513 | 9 | Exposed and functional residue |
| 24 | rs143172084 | R204C | 0.328 | 4 | Exposed |
| 25 | rs748161965 | E209K | -1.142 | 8 | Exposed and functional |
| 26 | rs201337537 | I210F | -1.225 | 9 | Buried and structural residue |
| 27 | rs774093278 | Y230C | -1.448 | 9 | Buried and structural residue |
| 28 | rs201932296 | H237Y | -1.437 | 9 | Buried and structural residue |
| 30 | rs762938662 | Y238C | -1.302 | 9 | Buried and structural residue |
| 29 | rs762938662 | Y238S | -1.302 | 9 | Buried and structural residue |
| 31 | rs543411711 | R246C | -0.551 | 7 | Exposed |
| 33 | rs780058774 | P249L | -0.9 | 8 | Exposed and functional residue |
| 32 | rs780058774 | P249R | -0.9 | 8 | Exposed and functional residue |
| 34 | rs753364550 | G262S | -1.46 | 9 | Buried and structural residue |
| 35 | rs765844957 | Q263R | -1.372 | 9 | Exposed and functional residue |
| 36 | rs772758672 | R264Q | -1.465 | 9 | Exposed and functional residue |
| 37 | rs774925080 | L267P | -0.841 | 7 | Exposed |
| 38 | rs568621983 | R274W | -1.093 | 8 | Exposed and functional residue |
| 39 | rs747922771 | L278P | -0.783 | 7 | Buried |
| 40 | rs765861589 | C281W | -1.512 | 9 | Buried and structural residue |

# Table S6: Structural assessment of ASTL homology model

| **ASTL** | **ALPHAFOLD2** | **I-TASSER** |
| --- | --- | --- |
| Ramachandran favored | 73.43% | 57.11% |
| Ramachandran outlier | 16.55% | 15.38% |
| Bad bonds | 1 | 18 |
| Bad angled | 91 | 181 |
| Clash score | 1.40 | 34.57 |
| QMEAN | 0.46 | 0.30 |
| MolProbity Score | 2.22 | 3.84 |

# Table S7: TM-align and RMSD values of the 40 mutant protein models

| **S.No** | **Variant ID** | **Substitution** | **TM Score** | **RMSD Values** |
| --- | --- | --- | --- | --- |
| 1 | rs765193573 | E75K | 0.885 | 3.89 |
| 2 | rs755524379 | K93N | 0.885 | 3.07 |
| 3 | rs150180534 | P105L | 0.857 | 3.71 |
| 4 | rs150180534 | P105R | 0.889 | 3.36 |
| 5 | rs774176117 | R117H | 0.842 | 3.65 |
| 6 | rs777987842 | T131M | 0.902 | 3.6 |
| 7 | rs145409944 | F135S | 0.806 | 4.65 |
| 8 | rs762760157 | G152R | 0.898 | 4.17 |
| 9 | rs759833663 | S155L | 0.844 | 4.21 |
| 10 | rs61735195 | R159H | 0.919 | 3.82 |
| 11 | rs774455538 | G162E | 0.802 | 4.26 |
| 12 | rs147042054 | H182L | 0.899 | 3.61 |
| 13 | rs147042054 | H182R | 0.885 | 3.68 |
| 14 | rs768755515 | L188P | 0.935 | 3.74 |
| 15 | rs746895908 | E193K | 0.821 | 4.2 |
| 16 | rs368781391 | R196W | 0.952 | 3.23 |
| 17 | rs780360443 | D198E | 0.811 | 4.65 |
| 18 | rs753572086 | D198N | 0.946 | 3.37 |
| 19 | rs753572086 | D198Y | 0.922 | 3.59 |
| 20 | rs750874250 | R199Q | 0.803 | 3.91 |
| 21 | rs151115044 | R199W | 0.847 | 4.35 |
| 22 | rs751579283 | D200N | 0.833 | 4.08 |
| 23 | rs751579283 | D200Y | 0.845 | 3.94 |
| 24 | rs143172084 | R204C | 0.95 | 3.08 |
| 25 | rs748161965 | E209K | 0.72 | 4.58 |
| 26 | rs201337537 | I210F | 0.9 | 3.71 |
| 27 | rs774093278 | Y230C | 0.887 | 3.95 |
| 28 | rs201932296 | H237Y | 0.881 | 3.56 |
| 29 | rs762938662 | Y238C | 0.93 | 3.39 |
| 30 | rs762938662 | Y238S | 0.885 | 3.9 |
| 31 | rs543411711 | R246C | 0.901 | 3.55 |
| 32 | rs780058774 | P249L | 0.902 | 3.6 |
| 33 | rs780058774 | P249R | 0.914 | 3.29 |
| 34 | rs753364550 | G262S | 0.868 | 3.74 |
| 35 | rs765844957 | Q263R | 0.896 | 3.63 |
| 36 | rs772758672 | R264Q | 0.81 | 4.14 |
| 37 | rs774925080 | L267P | 0.921 | 3.68 |
| 38 | rs568621983 | R274W | 0.854 | 3.68 |
| 39 | rs747922771 | L278P | 0.827 | 3.69 |
| 40 | rs765861589 | C281W | 0.845 | 4.19 |

# Caption for Supplementary Figures

**Figure SI 1** | A to H: Cluster of amino acid positions belonging to the eight independent sectors (IC1 to IC8) are shown in spheres.
